# On-Surface Azide–Alkyne Cycloaddition Reaction: Does It Click with Ruthenium Catalysts?

**DOI:** 10.1021/acs.langmuir.2c00100

**Published:** 2022-04-26

**Authors:** Tiexin Li, Essam M. Dief, Zlatica Kalužná, Melanie MacGregor, Cina Foroutan-Nejad, Nadim Darwish

**Affiliations:** † School of Molecular and Life Sciences, Curtin University, Bentley, Western Australia 6102, Australia; ‡ Institute of Organic Chemistry, Polish Academy of Sciences, Kasprzaka 44/52, 01-224Warsaw, Poland; § University of Warsaw, Faculty of Physics, Pasteura 5, 00-092Warsaw, Poland; ∥ Flinders Institute for Nanoscale Science & Technology, Flinders University, Bedford Park, South Australia5042, Australia; ⊥ Institute of Organic Chemistry and Biochemistry, Czech Academy of Sciences, Flemingovo nám. 2, CZ-16610Prague, Czech Republic

## Abstract

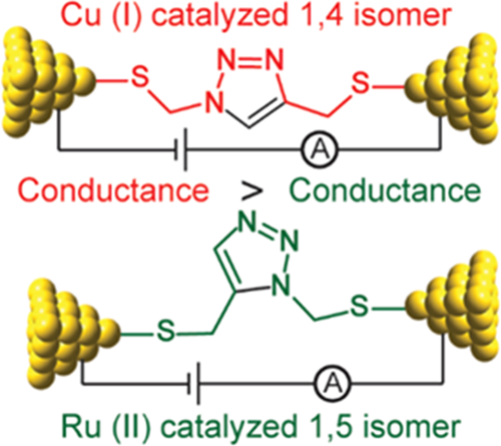

Owing to its simplicity, selectivity, high yield, and the absence of byproducts, the “click” azide–alkyne reaction is widely used in many areas. The reaction is usually catalyzed by copper(I), which selectively produces the 1,4-disubstituted 1,2,3-triazole regioisomer. Ruthenium-based catalysts were later developed to selectively produce the opposite regioselectivity—the 1,5-disubstituted 1,2,3-triazole isomer. Ruthenium-based catalysis, however, remains only tested for click reactions in solution, and the suitability of ruthenium catalysts for surface-based click reactions remains unknown. Also unknown are the electrical properties of the 1,4- and 1,5-regioisomers, and to measure them, both isomers need to be assembled on the electrode surface. Here, we test whether ruthenium catalysts can be used to catalyze surface azide–alkyne reactions to produce 1,5-disubstituted 1,2,3-triazole, and compare their electrochemical properties, in terms of surface coverages and electron transfer kinetics, to those of the compound formed by copper catalysis, 1,4-disubstituted 1,2,3-triazole isomer. Results show that ruthenium(II) complexes catalyze the click reaction on surfaces yielding the 1,5-disubstituted isomer, but the rate of the reaction is remarkably slower than that of the copper-catalyzed reaction, and this is related to the size of the catalyst involved as an intermediate in the reaction. The electron transfer rate constant (*k*_et_) for the ruthenium-catalyzed reaction is 30% of that measured for the copper-catalyzed 1,4-isomer. The lower conductivity of the 1,5-isomer is confirmed by performing nonequilibrium Green’s function computations on relevant model systems. These findings demonstrate the feasibility of ruthenium-based catalysis of surface click reactions and point toward an electrical method for detecting the isomers of click reactions.

## 1 Introduction

In 2001, Sharpless and co-workers developed “click” chemistry that enables the formation of 1,2,3-triazole compounds,^1^ and since then, the method has been widely used for synthetic chemistry.^2−6^ The reaction has two product possibilities: 1,4-disubstituted and 1,5-disubstituted triazoles. Different catalysts can yield either regioisomers or a mixture of both.^7−12^ The two isomers possess completely different chemical properties in solution and thus have different applications.^13−18^

Copper(I) is the most common catalyst used for the azide–alkyne cycloaddition, which is often referred to as the copper-catalyzed azide–alkyne cycloaddition (CuAAC) reaction. The CuAAC reaction has been an important advancement in the chemistry of 1,2,3-triazoles with an unprecedented reaction acceleration rate of 10^7^–10^8^ times higher than the uncatalyzed reaction in solution.^19^ More recently, pentamethylcyclopentadienyl ruthenium chloride [Cp*RuCl] complexes were discovered to catalyze the cycloaddition of azides to terminal alkynes in solution leading to 1,5-disubstituted 1,2,3-triazoles.^20,21^ In contrast to CuAAC reactions, the ruthenium-catalyzed azide–alkyne cycloaddition (RuAAC) reaction can also be used with internal alkynes, providing fully substituted 1,2,3-triazoles.

In parallel with advancement in azide–alkyne click reactions in solution, click reactions on surfaces became important for a range of applications including biological,^22^ pharmaceutical,^23−25^ and material science.^3,26−29^ To the best of our knowledge, all surface-based click reactions, to date, are based on copper Cu(I) catalysis, which produces exclusively the 1,4-isomer. It is not known whether achieving the 1,5-isomer selectively using ruthenium-based catalysts is possible on surfaces. Controlling and understanding the electrical properties of each isomer, specifically on surfaces, are important from the perspective of molecular devices and could help in identifying the isomers that are produced in other types of catalysis such as the oriented electric field catalysis^30−35^ and photoinduced click reactions.^36−38^

In this article, we test the feasibility of RuAAC reactions on surfaces. For this purpose, monolayers formed from 1,8-nonadiyne on Si surfaces were fabricated via a hydrosilylation reaction followed by reacting the terminal alkyne with azidomethylferrocene.^39−41^ The surface coverage and charge transfer kinetics were then studied using cyclic voltammetry (CV) and electrochemical impedance spectroscopy (EIS). Water contact angle measurements were used to monitor the exposed functional groups at the distal end of each monolayer, and X-ray photoelectron spectroscopy (XPS) was used to monitor the chemical functional groups on the surface and to assign the chemical transformations. The surface terraces were measured by atomic force microscopy (AFM). The impact of the size of the catalyst was studied using copper catalysts of different sizes, namely, Cu(I) by the reduction of CuSO_4_ by sodium ascorbate and bromotris(triphenylphosphine)copper(I). The structure of the monolayers was studied using density functional theory (DFT) calculations, while electron transport across the 1,4- and 1,5-triazole rings was studied using nonequilibrium Green’s functions.

## 2 Materials and Methods

### 2.1 Materials

Unless specified otherwise, all chemicals were of analytical grade and used as received. Chemicals used in surface modification and electrochemical experiments were of high purity (>99%). Milli-Q water (>18 MΩ cm) was used for surface cleaning, glassware cleaning, and the preparation of solutions. Dichloromethane (DCM) and 2-propanol were distilled before use. Hydrogen peroxide (30 wt % in water), sulfuric acid (Puranal TM, 95–97%), and ammonium fluoride (Puranal TM, 40 wt % in water) were of semiconductor grade and were used for Si wafer cleaning and etching. 1,8-Nonadiyne (98%) was obtained from Sigma-Aldrich and was used as received. Azidomethylferrocene was synthesized from ferrocene methanol using a procedure from the literature.^42^ Bromotris(triphenylphosphine)copper(I) (98%) and tris (2,2′-bipyridine) ruthenium(II) hexafluorophosphate (97%) were obtained from Sigma-Aldrich and used as received. Aqueous perchloric acid (1.0 M) was used as the electrolyte in all electrochemical measurements. Si wafers purchased from Siltronix, S.A.S. (Archamps, France), were p-type boron-doped with a thickness of 500 ± 25 μm and a resistivity of 0.007–0.013 Ω cm.

### 2.2 Surface Modification

#### 2.2.1 Silicon Passivation

The hydrosilylation reaction of 1,8-nonadiyne with Si–H followed a previously reported procedure.^42,43^ In brief, Si wafers were cut into pieces (approximately 1 × 1 cm^2^), cleaned in hot Piranha solution (130 °C, 3:1(v/v) mixture of concentrated sulfuric acid to 30% hydrogen peroxide) for 20 min, then rinsed with Milli-Q water, and etched in deoxygenated aqueous ammonium fluoride solution (40 wt %) under a stream of argon for 13 min. At this stage, the surface formed is hydrogen-terminated Si–H surface.^44−47^ The etched samples were rinsed with Milli-Q water and DCM before being placed in a deoxygenated sample of 1,8-nonadiyne. The surfaces were then rapidly transferred to a reaction chamber kept under nitrogen flow, and illuminated with UV light (Vilber, VL-215.M, λ = 312 nm) for 2 h.

#### 2.2.2 Copper-Catalyzed Azide–Alkyne Click Reaction (CuAAC)

**Figure 1:**
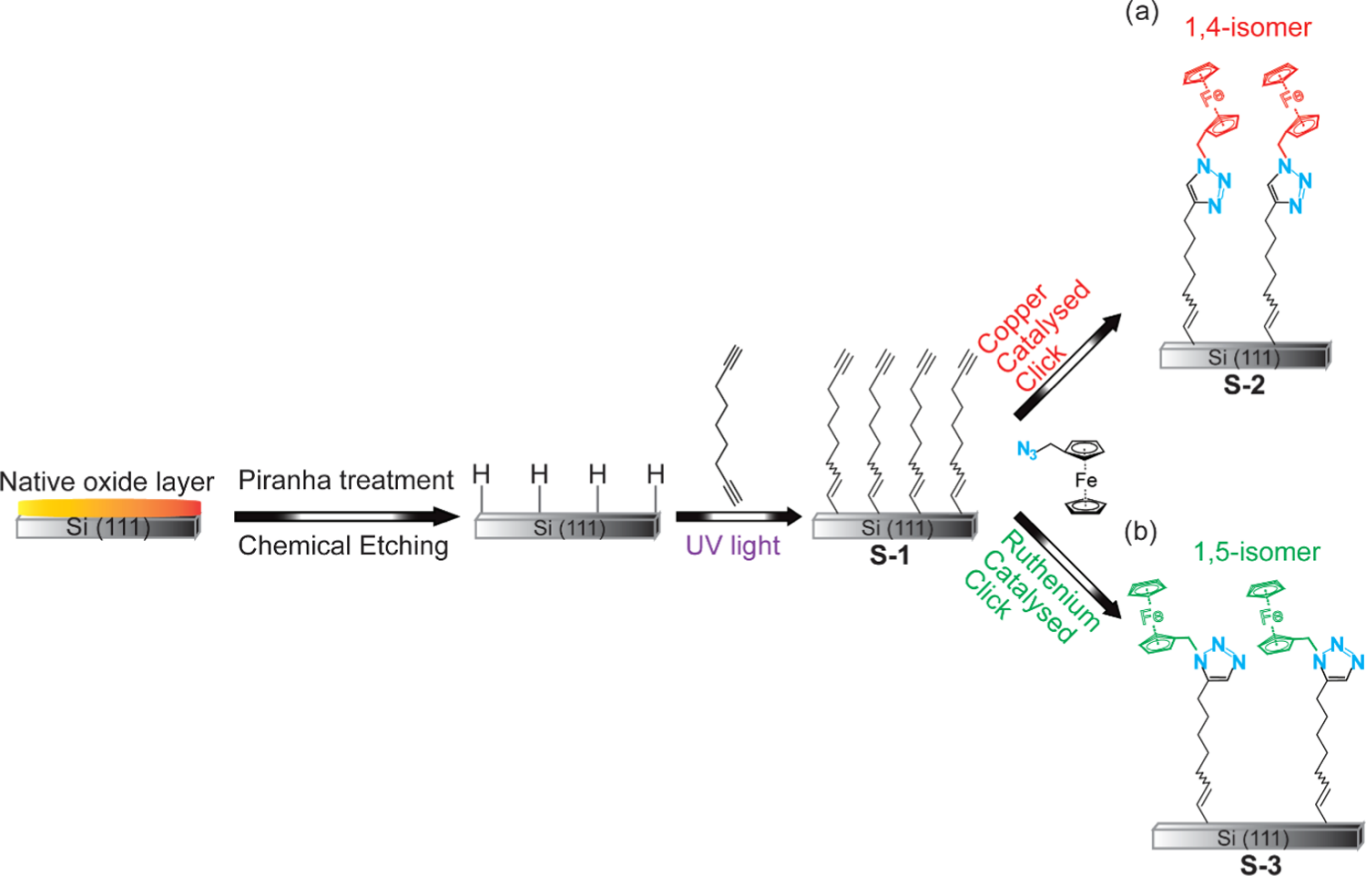
Schematic of the SAMs studied. Oxide-free silicon (Si–H) electrodes are reacted with 1,8-nonadiyne via a hydrosilylation reaction to form SAM **S-1**. A ferrocene moiety is attached to the distal end of the monolayer by (a) CuAAC reaction to yield the redox-active SAM **S-2** and (b) RuAAC reaction to yield the redox-active SAM **S-3**.

1,8-Nonadiyne SAMs (**S-1**, Figure 1) were reacted with azidomethylferrocene through a CuAAC reaction. In brief, **S-1** samples were incubated in a solution of 0.4 μM copper(II) sulfate pentahydrate, sodium ascorbate (5 mg/mL), and 0.5 mM azidomethylferrocene, under dark conditions. The reaction time was 120 min at room temperature. The Si electrodes were then removed from the solution and washed sequentially with 2-propanol, Milli-Q water, 0.5 M hydrochloric acid, Milli-Q water, 2-propanol, and DCM. Finally, the Si electrodes (**S-2**) were blown dry with a stream of argon before analysis.

#### 2.2.3 Ruthenium-Catalyzed Azide–Alkyne Click Reaction (RuAAC)

1,8-Nonadiyne SAMs (**S-1**, Figure 1) were reacted with azidomethylferrocene through an RuAAC reaction. In brief, **S-1** samples were incubated in a solution of 0.4 μM tris (2,2′-bipyridine) ruthenium(II) hexafluorophosphate, in toluene,^48,49^ and 0.5 mM azidomethylferrocene, under dark conditions. The reaction time was 72 h at room temperature. The silicon substrates were then removed from the solution and washed with toluene. Finally, the Si electrodes (**S-3**) were blown dry with a stream of argon before analysis.

### 2.3 Surface Characterization

#### 2.3.1 Electrochemical Measurements

Electrochemical measurements were carried out in a single-compartment, three-electrode poly(tetrafluoroethylene) (PTFE) cell using a CHI650 electrochemical workstation (CH Instruments). The modified Si surface (**S-2** and **S-3**) served as the working electrode, a platinum wire as the auxiliary electrode, and an Ag/AgCl aqueous electrode (1.0 M KCl, CH Instruments) as the reference electrode. Aqueous 1.0 M perchloric acid was used as the electrolyte. The electrical contact between the Si and the copper plate was reached by rapidly rubbing gallium indium eutectic on the back side of the Si electrode. EIS measurements were carried out at a DC offset equal to the half-wave potential (*E*_1/2_) measured in the CV experiments.^47,50−53^ The AC amplitude was 15 mV, and the frequency was scanned between 1 and 100,000 Hz. The surface coverages (Γ) of ferrocene molecules were calculated from the integration of the CV oxidation and reduction waves according to Γ = *Q*/*nFA* (where *Q* is the charge, *n* is the number of electron transfer, *F* is Faraday constant, and *A* is the area of electrode).

#### 2.3.2 Contact Angle Analysis

The wettability of the Si surfaces was measured by an automated static water contact angle with a Krüss DSA 100 goniometer. The reported values are the average of the angle between droplets and the surface, and the error bars represent the standard deviation of angle measurement of three different droplets on three different surfaces.

#### 2.3.3 Atomic Force Microscopy (AFM)

All topography images were conducted on a Bruker Dimension FastScan atomic force microscope in air and at room temperature. All of the AFM data were processed with NanoScope Analysis. Antimony (*n*)-doped silicon tips (TESPA-V2, Bruker AFM Probes), with a spring constant of 42 N/m and a resonance frequency of 320 kHz, were used as the AFM tips. The measurements were performed in tapping mode, and the size of the image was set to 5 × 5 μm^2^. The resolution was set to 256 points/line, and the scan rate was set to 1.0 Hz.

#### 2.3.4 X-ray Photoelectron Spectroscopy (XPS)

X-ray photoelectron spectroscopy (XPS) analysis of the monolayer-modified silicon surfaces was performed on a Kratos Axis Ultra DLD fitted with a monochromatic Al Kα (hυ1486.6 eV) radiation source operating at 225 W, and a hemispherical analyzer (165 mm radius) running in fixed-analyzer transmission mode. The photoelectron take-off angle was normal to the sample, and the chamber was operated at 2 × 10^–8^ Torr. The analysis area was 300 × 700 μm^2^, and an internal flood gun was used to minimize sample charging. Survey spectra (accumulation of three scans) were acquired between 0 and 1100 eV, with a dwell time of 55 ms, a pass energy of 160 eV, and a step size of 0.5 eV. High-resolution scans (accumulation of 10 scans) used a pass energy of 20 eV and a step size of either 0.05 eV (Si 2p, 90–110 eV) or 0.1 eV (C 1s, 277–300 eV). XPS data were processed in CasaXPS (version 2.3.18), and any residual charging was corrected by applying a rigid shift to bring the main C 1s emission (C–C) to 284.7 eV.

### 2.4 Computational Methods

**Figure 2:**
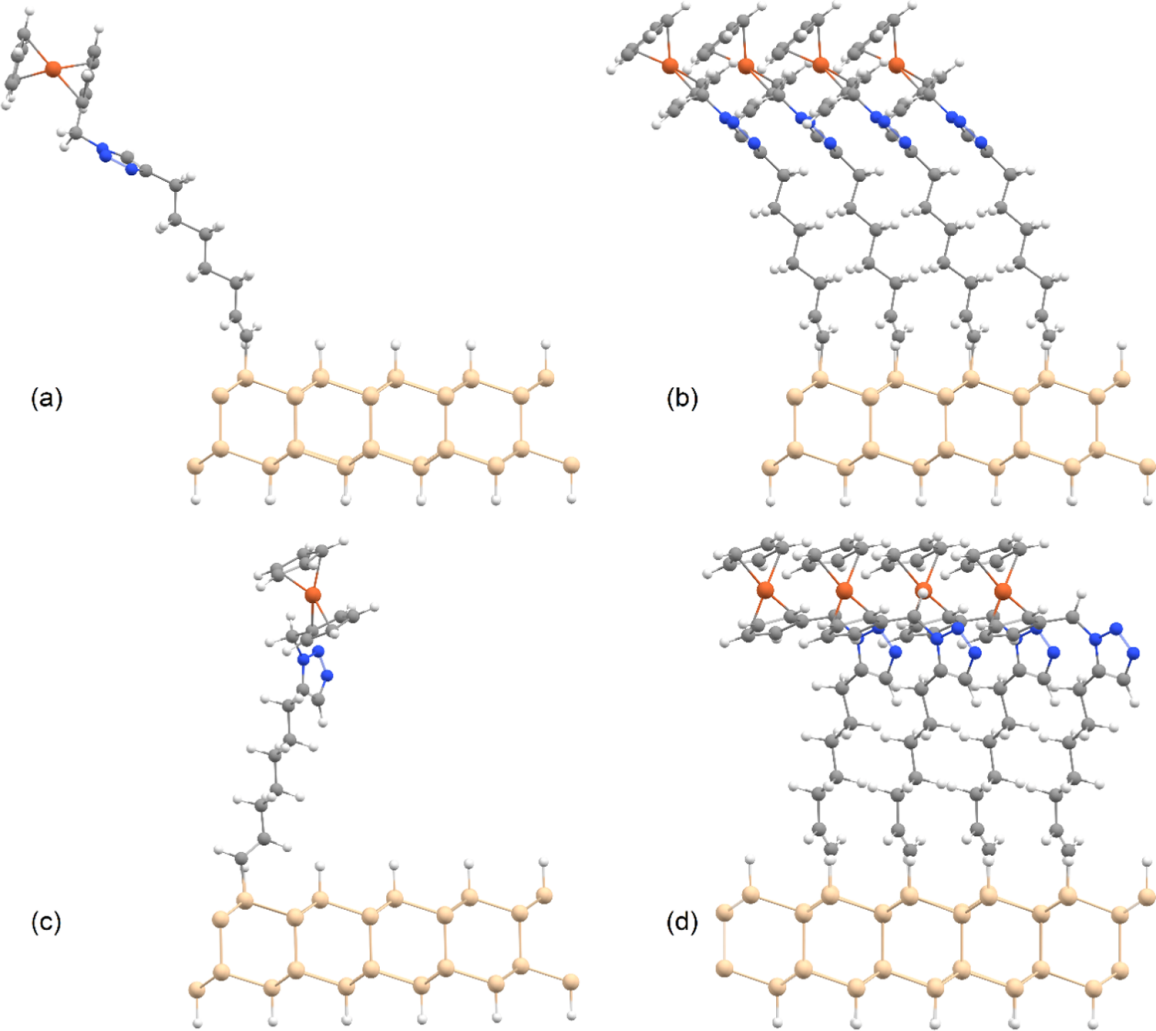
Most stable conformations of (a, b) 1,4-isomer and (c, d) 1,5-isomer on silicon surface. (a, c) Sparsely substituted Si surface. (b, d) Conformations of the isomers on a densely substituted Si surface. See the Methods section for computational details.

To identify the most stable conformation of substituted alkynes on Si surface, monolayers of alkyne, 1,4- and 1,5-isomers on silicon were modeled by the application of periodic boundary conditions on two different units cells containing different numbers of Si, H atoms, and the related molecules via generalized gradient approximation (GGA) functional developed by Perdew, Burke, and Ernzerhof (PBE)^54,55^ in combination with def2-SVP^56,57^ basis set by Gaussian 16.^58^ A small unit containing 15 Si, 7 H, and the molecules were modeled, which corresponds to a surface in which 25% of Si atoms on the surface are connected to 1,4- or 1,5-isomers. In this model, the alkynes are tightly packed. A less tightly packed surface with only 6.25% Si coverage was modeled, in which substituted alkynes are with a good estimation isolated on the surface without interacting with each other to identify the conformational changes of the head of the molecule upon close packing. Figure 2 displays the differences in the conformations of the species in tightly packed as well as the sparsely covered surfaces. For Cartesian coordinates, see the Supporting Information. Only the most stable conformation of the alkane chain, i.e., all anti, was considered for modeling. Although the alkane chains are flexible in gas or liquid phases, it is known that in SAMs, the close contact between molecules keeps the alkanes in a mostly organized structure akin to a crystal according to a recent study by Whitesides and his co-workers.^59^ The conformation of the head part of the molecules was thoroughly analyzed by scanning the dihedral angles around the CH_2_–C_(triazole)_, N_(triazole)_–CH_2_, and CH_2_–C_(Cp)_ bonds in 60 degrees to find the most stable conformation. In the surface-decorated species, only one stable conformation for each of the 1,4- and 1,5-isomers was identified, and they are presented below. The reason for the absence of the other isomers is likely the repulsion between neighboring molecules in SAM that pushes the head part and fixes it in one low-energy conformation. The depiction of the van der Waals radii of atoms in the molecules clearly shows the proximity of the ferrocene heads on the silicon surface together that enforces a certain conformation to the molecules (Figure S1, Supporting Information).

To assess the conductivity of 1,4- and 1,5-isomers, two model systems with 1,4- and 1,5-thioethynyl substituents on 1,2,3-triazole moiety were optimized at the B3LYP^60−62^/def2-TZVPP^56^ computational level by Gaussian 16 software. Nonequilibrium Green’s function (NEGF) computations coupled with DFT were employed to assess the intrinsic conductivity of 1,4- and 1,5-isomers of 1,2,3-triazole. Our systems were dissected into three regions, which consist of a scattering region and two semi-infinite electrodes. No direct interaction between the electrodes was considered. The Au(001) surface of a bulk gold structure was selected to utilize the electrodes. Each electrode consists of five and four gold layers in sequence, and a single Au atom as the tip of the electrode. The preoptimized 1,4- and 1,5-isomers were embedded between two gold atoms at the tips of the electrodes via the sulfur atoms of thioethynyl groups by setting the Au–S distance fixed to 2.3 Å. DFT-NEGF computations were performed by TranSiesta module^63^ using PBE functional with double-ζ polarized basis set as implemented in Siesta suite of programs. The energy cutoff was set to 300 Ry for the real space grid. Γ points for sampling were used for the first Brillouin zone in the molecular region and 1 × 1 × 100 Monkhorst–Pack *k*-point grid for the nanowire electrodes.

## 3 Results and Discussion

### 3.1 Surface Characterization

**Figure 3:**
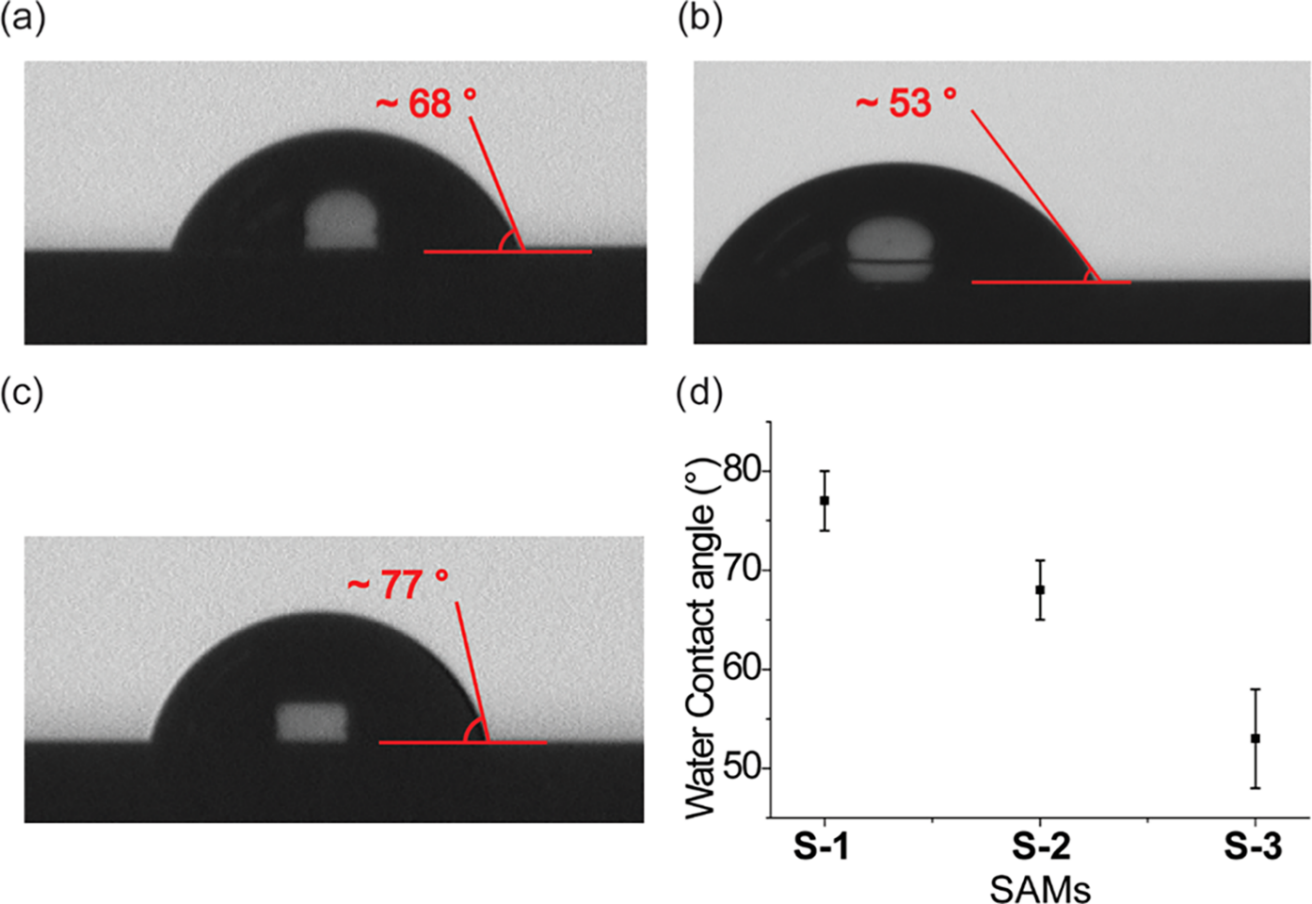
Static image of a water droplet on (a) SAM **S-2**, which was formed by a CuAAC reaction; (b) SAM **S-3**, which was formed by a RuAAC reaction; and (c) SAM **S-1** before the click reaction. (d) Water contact angles for SAM **S-1**, **S-2**, and **S-3**. The error bars in (d) are the standard deviation of water contact angles from the mean value of three different surfaces.

The surfaces were first analyzed using water contact angles to detect differences in the functional groups at the distal end of each monolayer. Figure 3d shows that the water contact angle for the 1,4-disubstituted 1,2,3-triazole isomer (SAM **S-2**) is 68°, while that for the 1,5-disubstituted 1,2,3-triazole isomer (SAM **S-3**) is 53°. This is likely because the 1,4-isomer forces the ferrocene moieties to face upward and the triazole rings are parallel to the Si surface such that the nitrogen atoms in the triazole ring are not exposed to the water droplet (Figures 2a,c and 3a). On the other hand, the 1,5-isomer of the RuAAC reaction forces the ferrocene moieties away from the nitrogen atoms of triazoles that are perpendicular to the Si surface and are comparatively more exposed to the water droplet leading to an increase in the surface wettability (Figures 2b,d and 3b). As a reference, the contact angle of the 1,8-nonadiyne SAM **S-1**, without the triazole ring-bound ferrocene, showed a contact angle of 77° (Figure 3c), whereas SAM **S-1** incubated in the ferrocene azide solution without catalysts showed a contact angle of 74° similar to that observed for SAM **S-1** before the hydrosilylation reaction (Figure S2, Supporting Information).

**Figure 4:**
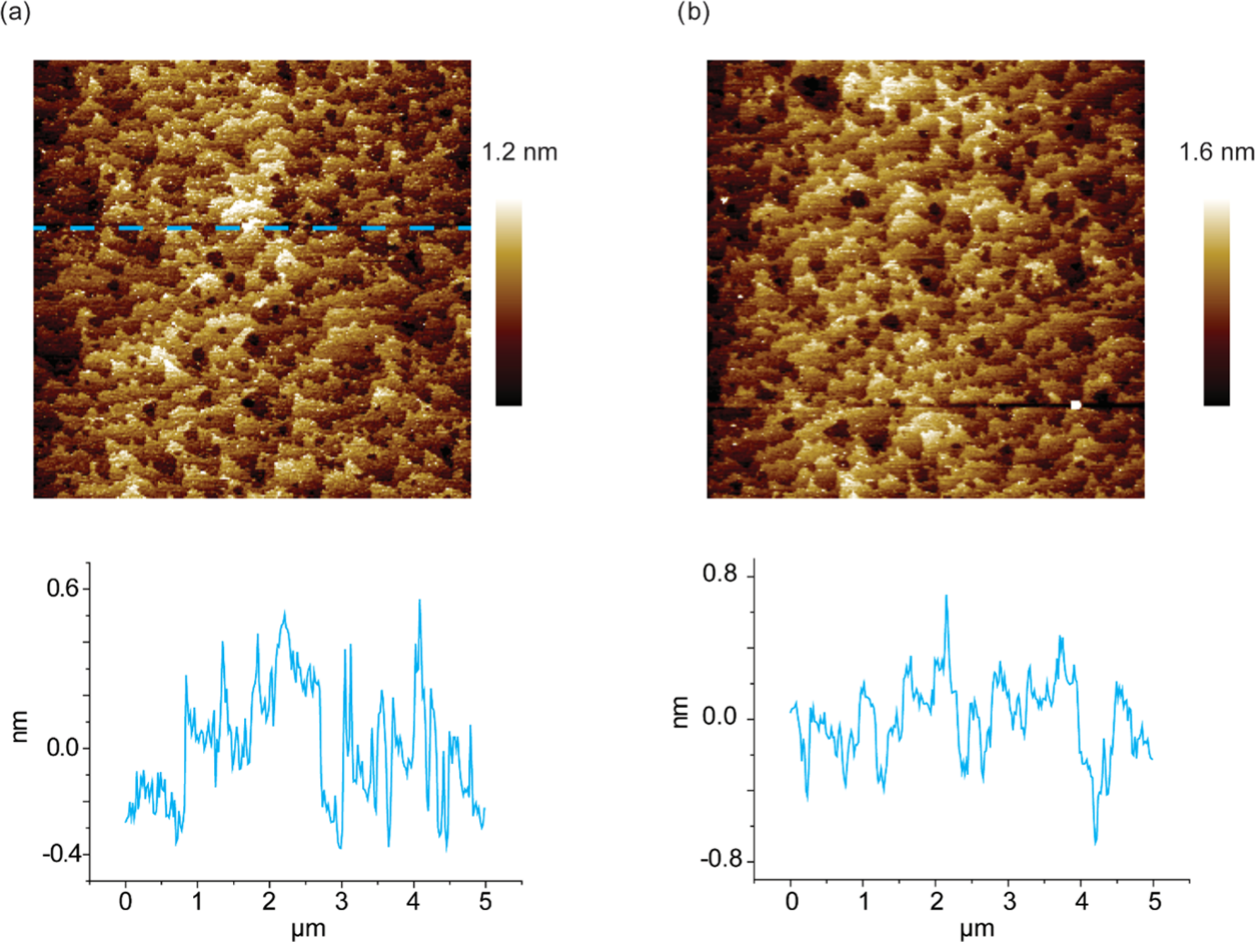
AFM topography images of (a) SAM **S-2**, which was formed by CuAAC reaction, and (b) SAM **S-3**, which was formed by RuAAC reaction. The insets in (a) and (b) show cross-sectional profile (line) roughness.

The AFM images for SAM **S-2** and **S-3** show clearly visible flat terraces with smooth edges (Figure 4a,b). The root-mean-square roughness (RMS) for SAM **S-2** is 0.275 nm, while that for SAM **S-3** is 0.306 nm. The roughness is within the range of typical monolayers on Si^64^ and indicates that the structure and the oxide content of both surfaces are similar.

**Figure 5:**
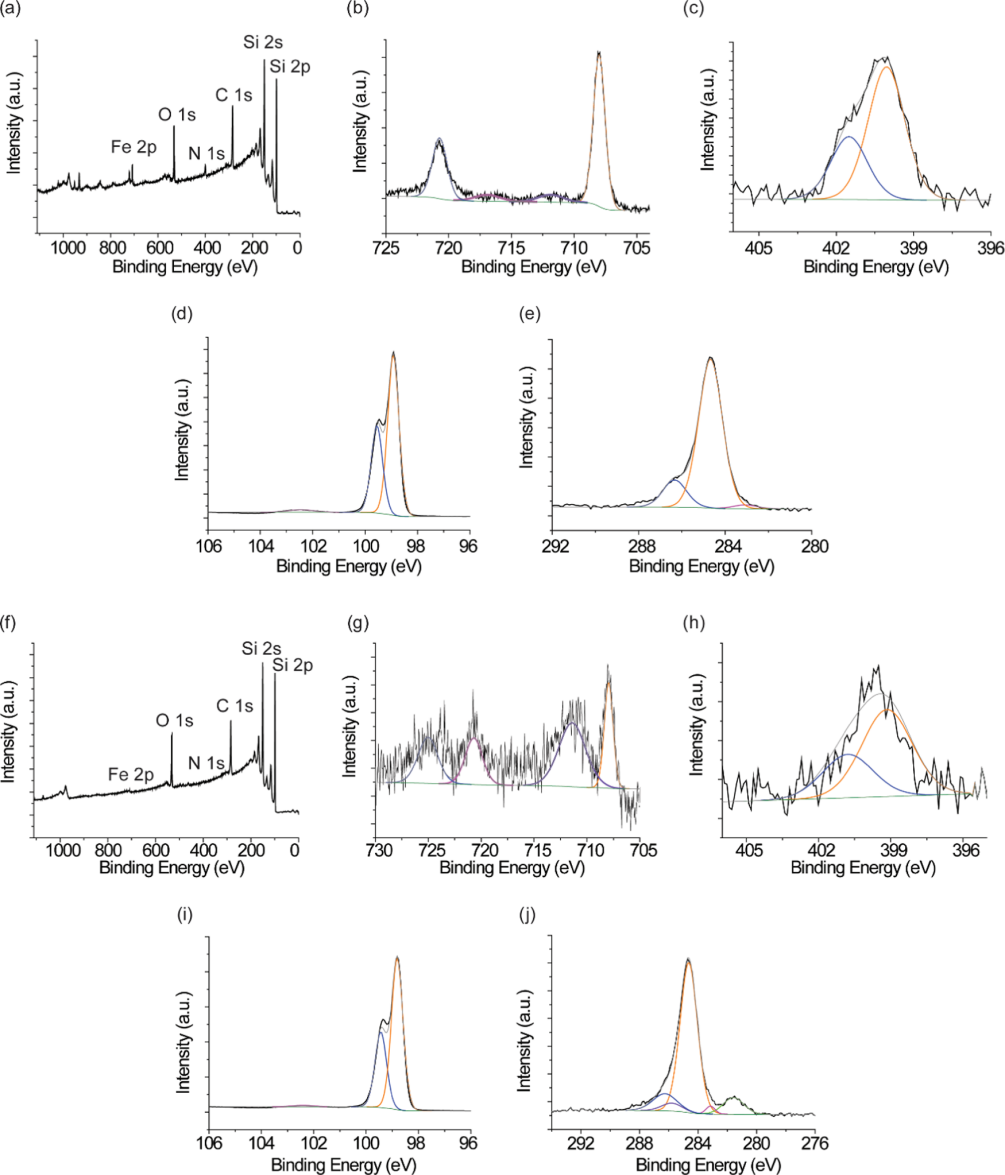
(a) XPS survey spectra of the monolayer of **S-2** SAM. XPS high-resolution spectra for **S-2** SAM for (b) Fe 2p, (c) N 1s, (d) Si 2p, and (e) C 1s. (f) XPS survey spectra of the monolayer of **S-3** SAM with a reaction time of 24 h. XPS high-resolution spectra of **S-3** SAM for (g) Fe 2p, (h) N 1s, (i) Si 2p, and (j) C 1s for which the reaction time was 24 h.

XPS shows the expected signals from SAM **S-2** and **S-3** (Figure 5). For the CuAAC reaction (Figure 5a–e), XPS Fe 2p narrow scan of surface **S-2** shows clear Fe emission. The Fe 2p high-resolution envelope was fitted by four peaks. The two peaks at 708.09 and 720.84 eV correspond to Fe 2p_3/2_ and Fe 2p_1/2_ for low- and high-energy spins, respectively. The two emissions at 711.91 and 717.06 eV are satellite peaks for Fe(II) Fe 2p_3/2_ and Fe(III) Fe 2p_3/2_, respectively (Figure 5b). The high-resolution N 1s spectra showed a broad peak centered at ∼400.5 eV, suggesting the presence of chemically distinct nitrogen atoms consistent with the formation of a triazole moiety. The best-fitting peaks at 400.02 and 401.52 eV, correspond to N–N bonding and N=N bonding, respectively. If any unreacted azide species was physically absorbed in the monolayer, then a well-resolved peak at ∼405 eV corresponding to the electron-deficient nitrogen in the azide group would be expected. No such peak was observed in our spectra (Figure 5c). For the RuAAC reaction performed for 24 h (Figure 5f–j), the Fe 2p narrow scan of surface **S-3** shows a weak Fe signal. Both Fe 2p and N 1s signals are small (Figure 5g,h), but consistent with that obtained for the CuAAC reaction. Again for the RuAAC reaction, no emission was observed in the spectra at ∼405 eV, indicating no physical adsorption of unreacted azide species present in the monolayer (Figure 5h). The presence of emission at ∼400.5 eV is an indication that the azide transformation to triazole has been achieved for both CuAAC and RuAAC reactions. It should be noted that the Fe/Si ratio is significantly higher for the CuAAC reaction (CuSO_4_ and sodium ascorbate) than the RuAAC. For the CuAAC reaction, the ratio is 0.57%, while the ratio is only 0.1% for the RuAAC reaction for 24 h (Table S1, Supporting Information). The lower Fe/Si ratio for the RuAAC-catalyzed surface is also reasonable considering the higher coverages observed for the CuAAC reaction in the electrochemical measurements.

### 3.2 Electrochemical Characterization

**Figure 6:**
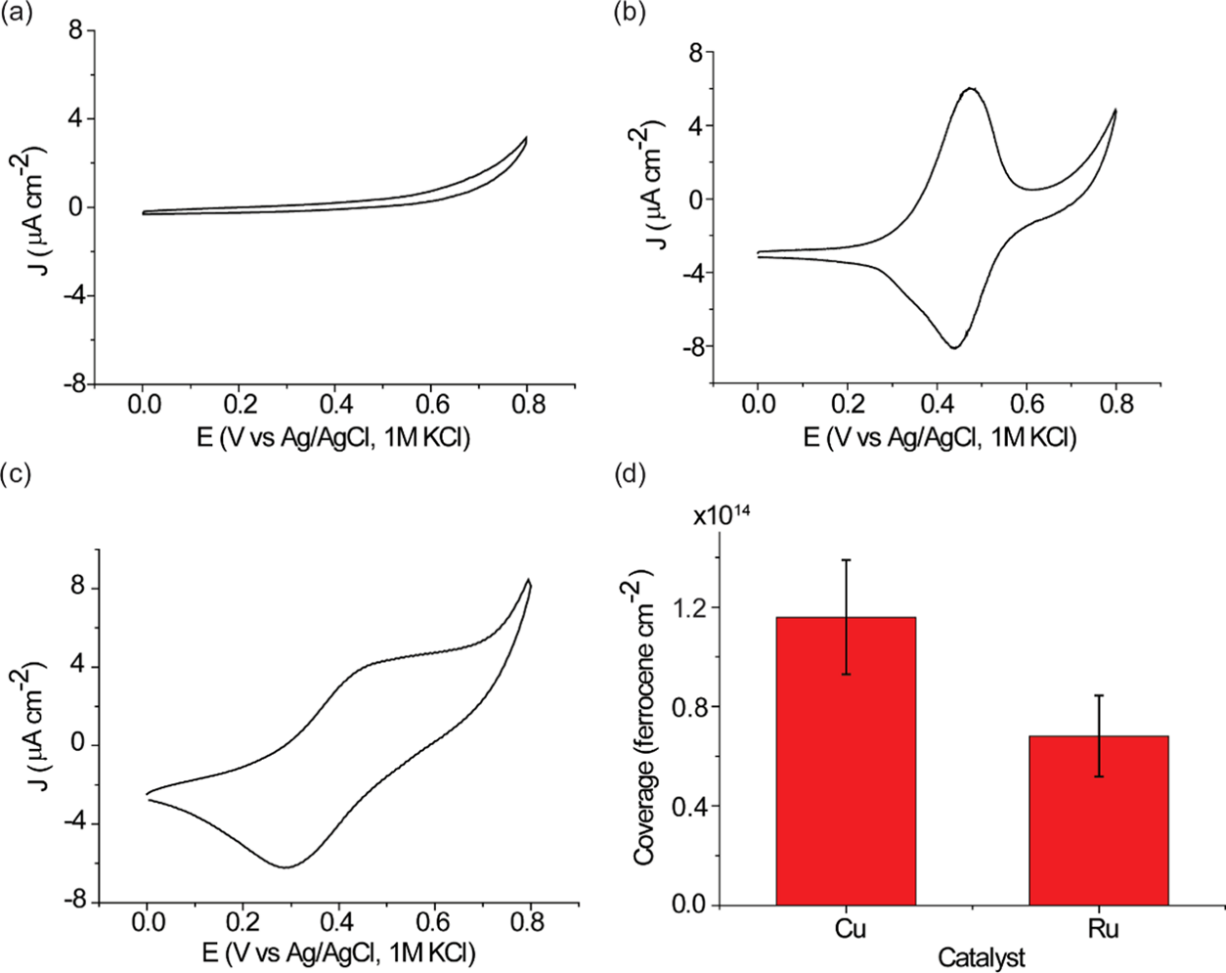
Electrochemical characterization of SAM **S-2** and **S-3**, which were formed by CuAAC and RuAAC reactions, respectively. CVs for (a) a click reaction without any catalyst; (b) SAM **S-2**, which was formed by the CuAAC reaction; and (c) SAM **S-3**, which was formed by the RuAAC reaction at the scan rate of 0.1 V/s. (d) Corresponding surface coverages calculated from the oxidation waves of the CVs in (b) and (c). The error bars in (d) are the standard deviation of the surface coverages obtained from the mean value of three different surfaces.

The surfaces were then analyzed electrochemically. Figure 6a shows the CVs for SAM **S-2** with the typical redox signal of a surface-bound ferrocene moiety catalyzed by the CuAAC reaction.^52,65,66^ The oxidation and reduction are nearly symmetrical with an *E*_1/2_ centered at 0.46 V. The separation of the oxidation and reduction peaks is only 33 mV. In comparison, the CV waves for SAM **S-3**, which was catalyzed via a RuAAC reaction, are separated by 190 mV (Figure 6b,c). The difference in peak separation at the same scan rate is likely due to the difference in the electron transfer kinetics.^53,65^ The surface coverage is (1.16 ± 0.23) × 10^14^ ferrocene cm^–2^ and (7.92 ± 1.90) × 10^13^ ferrocene cm^–2^ for the CuAAC and RuAAC reactions, respectively (Figure 6d). Performing the reaction without a catalyst led to no detectable redox signals (Figure 6a). The CVs for different scan rates show a linear relationship between the faradaic peak current and scan rate (Figure S3, Supporting Information). The adjusted R-square values are all close to 1 for both oxidation and reduction peak current for SAM **S-2** and **S-3**. It is noted that the cathodic current density is larger than the anodic current density in SAM **S-3**. This can be explained by the position of the ferrocene moieties, in the case of RuAAC reaction, which are not as organized at the distal end of the monolayer as they are in SAM **S-2**. This agrees with the AFM images, which showed that SAM **S-3** is slightly rougher than SAM **S-2**. The surface coverage for the RuAAC-catalyzed 1,5-isomer is ∼60% of that measured for the 1,4-isomer despite a much longer reaction time (72 and 2 h for RuAAC and CuAAC reactions, respectively). Despite the reactivity differences, the RuAAC reaction offers a new isomeric form, the 1,5-disubstituted 1,2,3-triazole, which can be applied to surface click reactions in which the 1,5-isomers are desirable. It also offers an opportunity to perform surface azide–alkyne reactions without the use of Cu ions, which are not desirable for some applications.^67^ We also found that high temperatures can slightly offset the energy cost and steric hindrance involved in the formation of the 1,5-isomer by the RuAAC reaction. We performed the RuAAC reaction in different solvents at different temperatures (35, 65, and 85 °C) for 180 min. The results show that the coverage of the clicked ferrocene increases from (6.14 ± 1.66) × 10^12^ ferrocene cm^–2^ to (3.08 ± 0.86) × 10^13^ ferrocene cm^–2^ in the solvent of toluene when the temperature increases from 35 to 65 °C. The coverage then decreases to (5.89 ± 1.47) × 10^12^ ferrocene cm^–2^ when the temperature reaches 85 °C (Figure S4, Supporting Information). This is possibly because the monolayer partially desorbs or the ferrocene starts decomposing. Similar trends are observed for other solvents such as tetrahydrofuran, 1,2,3-trichlorobenzene, and mesitylene (Table S2, Supporting Information).

### 3.3 Investigating the Size of the Catalyst on the Yield of the Surface Reaction

To investigate the effect of the size of the catalysts, we used a bigger-size copper catalyst, bromotris(triphenylphosphine)copper(I). The CVs results show that after the reaction was carried out for 2 h, 1,4-disubstituted 1,2,3-triazole forms (Figure S5, Supporting Information) with coverage that is less than that obtained for a surface that was catalyzed by the smaller-sized Cu(I) catalyst (obtained by reduction of Cu(II) in CuSO_4_ by sodium ascorbate). For instance, in a period of 2 h, the Cu(I) catalyst yields (1.16 ± 0.23) × 10^14^ ferrocene cm^–2^, while the bulky copper catalyst yields (3.35 ± 0.70) × 10^13^ ferrocene cm^–2^, indicating faster kinetics with smaller catalysts. Therefore, the size of the catalysts appears to control the rate of RuAAC and CuAAC surface reactions. The effect of the size of the catalyst can be explained by the steric hindrance associated with the bulky catalysts, which is involved as an intermediate during the reaction. For instance, copper acetylide intermediates are suggested to form during the reactions’ catalytic cycle.^68,69^ It should be noted that high coverages can be achieved using the bulky copper catalyst but requires more time. For example, the coverage increases from (3.35 ± 0.70) × 10^13^ ferrocene cm^–2^ to (1.07 ± 0.27) × 10^14^ ferrocene cm^–2^ when the reaction time increases from 2 to 72 h using bromotris(triphenylphosphine)copper(I) catalysis.

### 3.4 Electron Transport Measurement and Modeling

**Figure 7:**
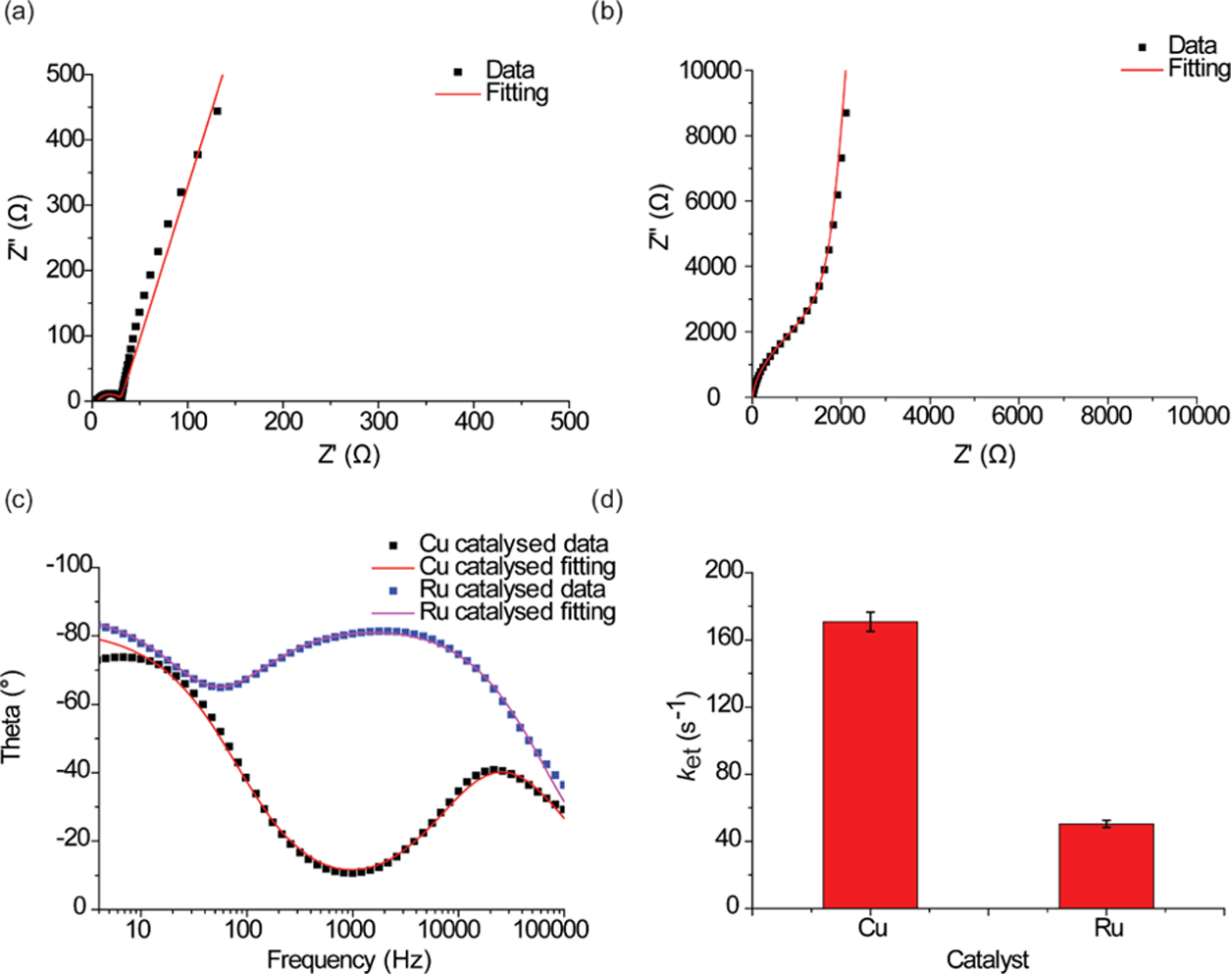
Nyquist plot from EIS measurement for (a) SAM **S-2** which was formed by CuAAC reaction, and (b) SAM **S-3**, which was formed by RuAAC reaction. (c) Bode plots for CuAAC and RuAAC reactions with a frequency range of 4–60,000 Hz. Scattered dots (black and blue) are experimental data and lines (red and magenta) that are best fit to the experimental data. (d) Evolution of *k*_et_ obtained by fitting the impedance data to a Randles circuit (Table S3, Supporting Information). The error bars in (a) are the standard deviation of *k*_et_ values obtained from the mean value of three different surfaces.

For the purpose of assessing the charge transfer kinetics, electrochemical impedance spectroscopy (EIS) was performed on the **S-2** and **S-3** SAMs. The electron transfer kinetics (*k*_et_) was 170.76 ± 5.73 and 50.43 ± 2.12 s^–1^ for SAM **S-2** and **S-3**, respectively (Figure 7a–d). The equivalent circuit used to fit the EIS data is shown in Figure S6 (Supporting Information). The difference in *k*_et_ values can be attributed to the difference in the electron transfer pathways across the disubstituted 1,4- and 1,5-triazole moieties.

**Figure 8:**
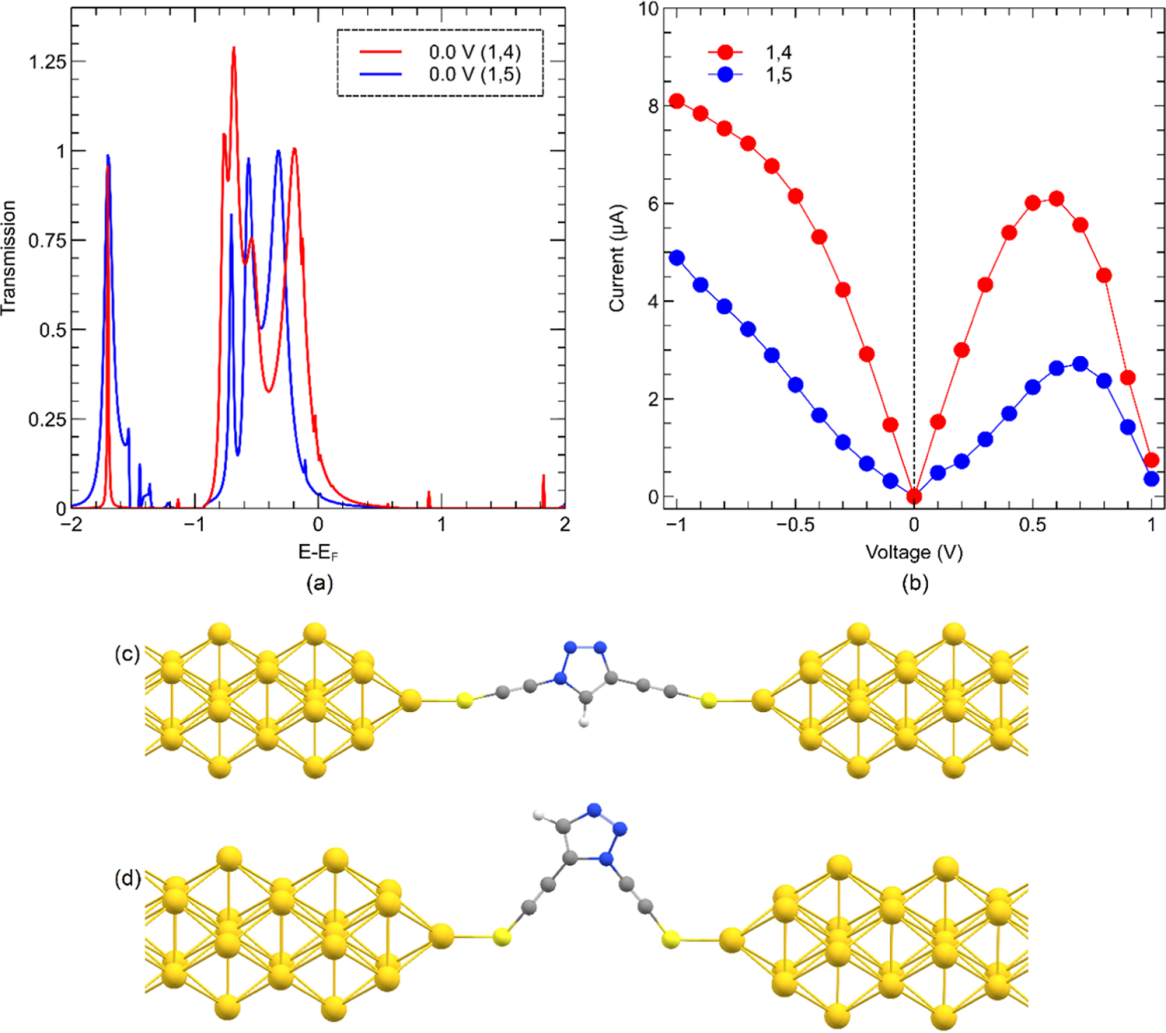
(a) Transmission spectra at zero-bias and (b) current–voltage characteristics of (c) 1,4- and (d) 1,5-isomers of 1,2,3-triazole model systems computed between two gold nanowires. Transmission spectra suggest that the main active orbitals in the conductance are the highest occupied molecular orbital (HOMO)-type orbitals that have energies lower than the Fermi level. The conductivity of the model systems is computed in a range of −1 to +1 V, and in the full range, the 1,4-isomer (red trace) remains more conductive than the 1,5-isomer (blue trace). The conductivity of the 1,4-isomer in the range of −0.1 to +0.1 V is computed to be 15 μS and that of the 1,5-isomer in the same range is predicted to be about 3.1–4.8 μS. Thus, the 1,5-isomer’s conductivity is ∼30% of that of the 1,4-isomer in the aforementioned voltage range.

To test this hypothesis, we performed NEGF on model molecules in which the two isomers are connected between two Au nanoelectrodes via two identical thioethynyl groups to eliminate the substituent effect on the conductivity. Figure 8a–d represents the electron transmission spectra of the two isomers in the same energy span and their current–voltage *I*/*V* curves in the range of an applied bias voltage between −1 and +1 V. As it is clear from the both plots, the 1,4-isomer has a higher intrinsic conductivity that is consistent with the experimental results.

## 4 Conclusions

In summary, we demonstrate that the RuAAC reaction is a feasible surface chemical reaction. Although the RuAAC reaction is sluggish compared to the CuAAC, they are nevertheless a viable click surface reactions for systems in which copper ions need to be avoided.^67^ It is demonstrated that the size of the catalyst controls the kinetics of the reaction, which explains the differences in the reaction rate of RuAAC and CuAAC surface-based reactions. The RuAAC reaction offers new opportunities for molecular electronics and single-molecule circuitry by forming triazole rings whose electron pathway differs from that obtained by the typical copper catalysts. The electron transfer rate constant across the 1,5-isomer is about 3-fold lower than that measured for the 1,4-isomer. The experimental observation is supported by nonequilibrium Green’s function computations that measured 3-fold lower conductivity across a model system representing the 1,5-isomer of 1,2,3-triazole. These findings point toward an electrical method for detecting the 1,4- and 1,5-isomers for a range of metal-complex, light-activated, and electric field catalyzed click reactions and provide new opportunities for molecular circuitry.

## Supporting Information Available

Detailed contact angle, AFM, XPS, and electrochemical analysis (PDF)
