# Supplementary material for: On-Surface Azide–Alkyne Cycloaddition Reaction: Does It Click with Ruthenium Catalysts?
Source: Langmuir. 2022 Apr 26;38(18):5532–41. doi: 10.1021/acs.langmuir.2c00100 (PMC9097529; doi:10.1021/acs.langmuir.2c00100)
Supplement: Supplementary file 1 — la2c00100_si_001.pdf [file la2c00100_si_001.pdf]

Supporting Information

On-Surface Azide–Alkyne Cycloaddition Reaction: Does it Click with Ruthenium Catalysts?

Tiexin Li[1], Essam M. Dief[1], Zlatica Kalužná[2,3], Melanie MacGregor[4], Cina Foroutan-Nejad[2,5]*, and Nadim Darwish[1]*

[1] School of Molecular and Life Sciences, Curtin University, Bentley, WA 6102, Australia

[2] Institute of Organic Chemistry, Polish Academy of Sciences, Kasprzaka44/52, 01-224 Warsaw, Poland

[3] University of Warsaw, Faculty of Physics, 00-092 Warsaw, Pasteura 5, Poland

[4] Flinders Institute for Nanoscale Science & Technology, Flinders University, Bedford Park, SA 5042, Australia

[5] Institute of Organic Chemistry and Biochemistry, Czech Academy of Sciences, Flemingovo nám. 2, CZ-16610, Prague, Czech Republic

* Corresponding author.

E-mail: nadim.darwish@curtin.edu.au (Nadim Darwish); canyslopus@yahoo.co.uk (Cina Foroutan-Nejad)

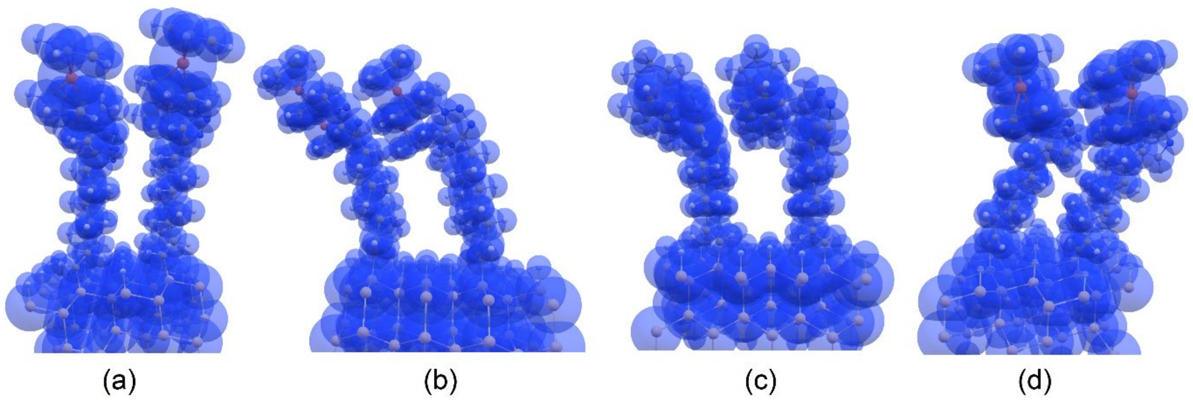

Figure S1. Representation of the van der Waals sphere of atoms in all atoms in the 1,4 and 1,5 isomers with 25% surface coverage. The head parts of the molecules are closely packed even at this rate of surface coverage.

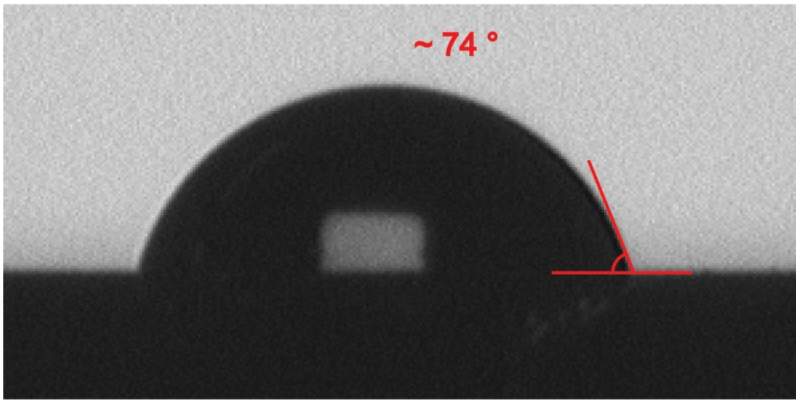

Figure S2. The static image of a water droplet on SAM **S-1** surface in which a nonadiyne SAM is immersed in the ferrocene azide solution in the absence of catalysts.

Table S1. The atomic percentage of Fe and Si in SAMs **S-2** and **S-3** obtained from XPS measurements. The percentage of Fe is calculated relative to Si.

SAMs	Fe %
**S-2**	0.57
**S-3** (24 hours)	0.10

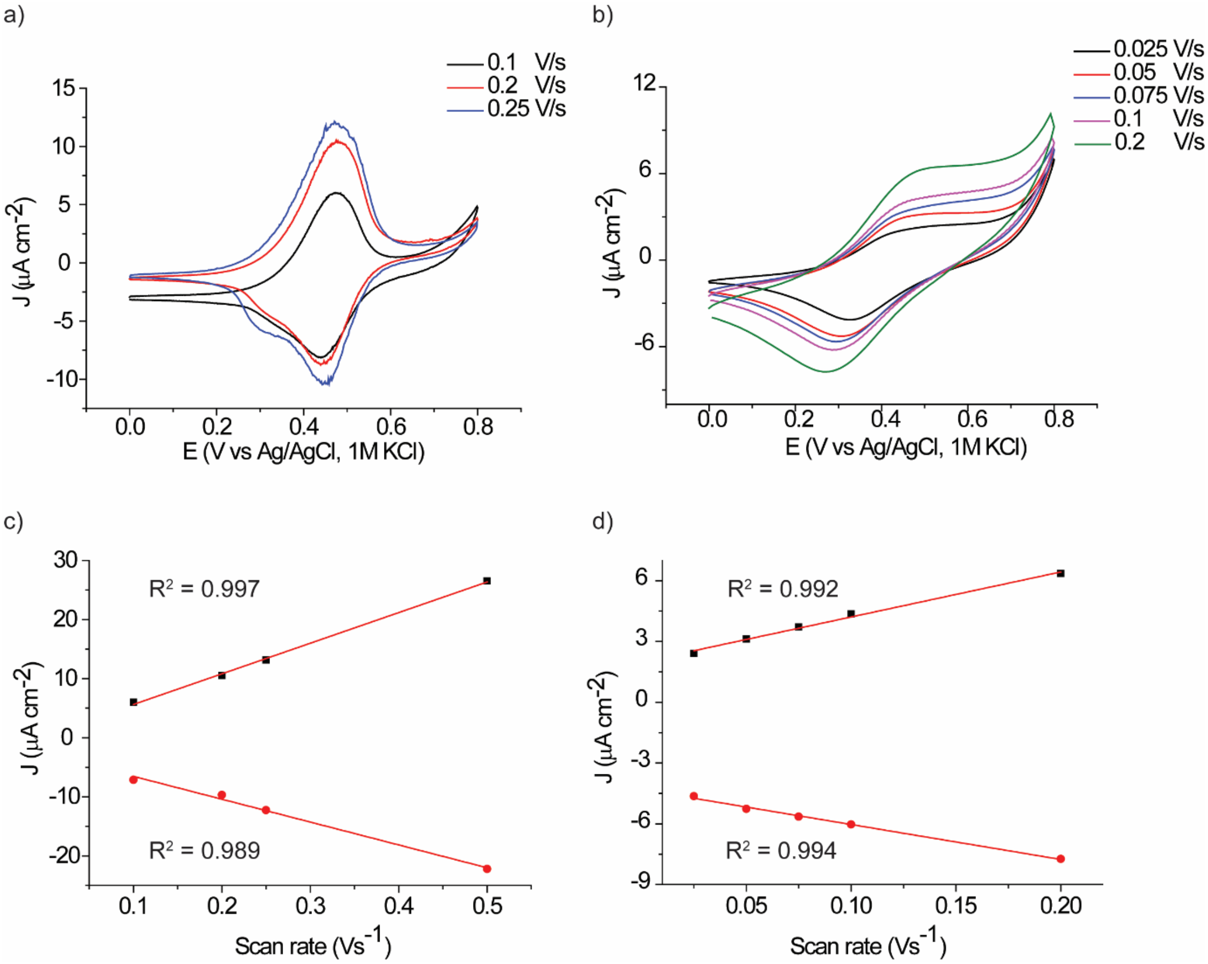

Figure S3. CVs for a) SAM **S-2** which was formed by CuAAC reaction, b) SAM **S-3** which was formed by RuAAC reaction at different scan rates. Peak current-density *vs* scan rates for c) SAM **S-2** which was formed by aCuAAC reaction, d) SAM **S-3** which was formed by RuAAC reaction.

.

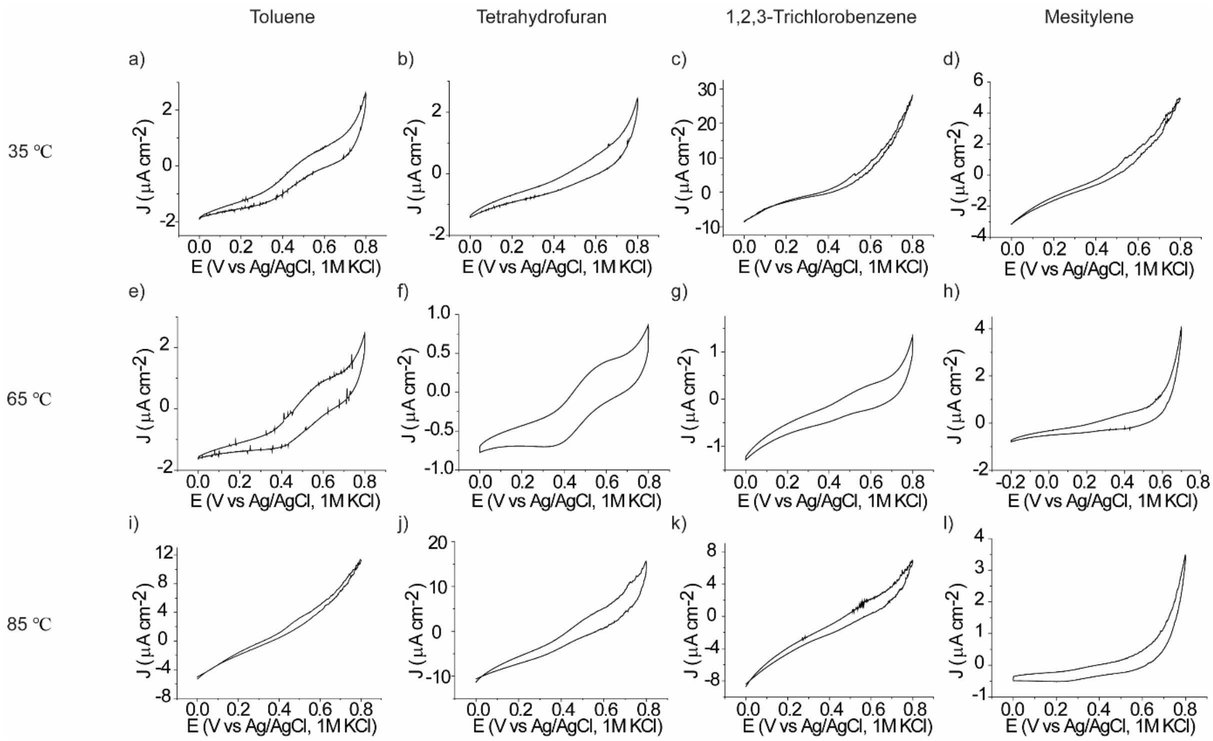

Figure S4. CVs for SAM **S-3** which was formed by RuAAC reaction at 35 °C for 180 minutes in a) toluene, b) tetrahydrofuran, c) 1,2,3-trichlorobenzene, and d) mesitylene. CVs for SAM **S-3** which was formed by RuAAC reaction at 65 °C for 180 minutes in e) toluene, f) tetrahydrofuran, g) 1,2,3-trichlorobenzene, and h) mesitylene. CVs for SAM **S-3** which was formed by RuAAC reaction at 85 °C for 180 minutes in i) toluene, j) tetrahydrofuran, k) 1,2,3-trichlorobenzene, and l) mesitylene.

Table S2. The surface coverage calculated from the oxidation waves of the CVs in Figure S4.

	Surface Coverage (ferrocene cm^{-2})			
	Toluene	Tetrahydrofuran	1,2,3-trichlorobenzene	Mesitylene
35 °C	$(6.14 \pm 1.66) \times 10^{12}$	$(2.35 \pm 0.59) \times 10^{12}$	$(6.71 \pm 1.74) \times 10^{11}$	$(8.52 \pm 1.96) \times 10^{11}$
65 °C	$(3.08 \pm 0.86) \times 10^{13}$	$(3.64 \pm 1.02) \times 10^{13}$	$(4.53 \pm 1.09) \times 10^{12}$	$(5.26 \pm 1.21) \times 10^{12}$
85 °C	$(5.89 \pm 1.47) \times 10^{12}$	$(6.74 \pm 1.95) \times 10^{12}$	$(3.47 \pm 0.90) \times 10^{12}$	$(1.96 \pm 0.55) \times 10^{12}$

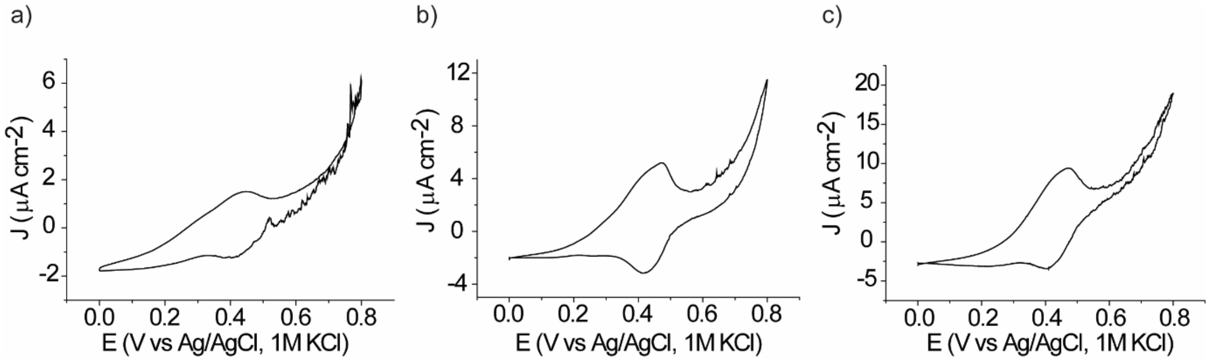

Figure S5. CVs for SAM **S-2** which was formed by CuAAC reaction and which was catalyzed by bromotris(triphenylphosphine)copper(I) for a) 2 h, b) 24 h, and c) 72 h.

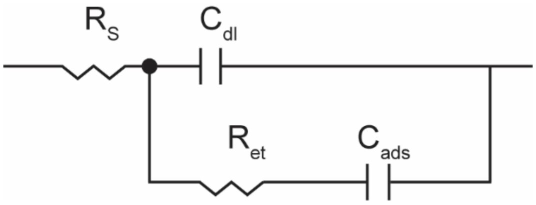

Figure S6. The Randles circuit used to fit the EIS data. The k_{et} is calculated according to the following equations:

$C_{dl} = (\frac{C}{A})(A)$

$C_{ads} = (\frac{n2F2A\Gamma}{4RT})$

$R_{ct} = (\frac{2RT}{n2F2A\Gamma Ket})$

In the above equations, C_{dl} is the double layer capacitance, C/A is the double layer capacitance per unit area, A is the electrode area, Γ is the coverage of the electroactive species per unit area, C_{ads} is the adsorption pseudocapacitance. R_{et} is the charge-transfer resistance, n is the number of electrons transferred. The electron transfer rate constant is calculated using:

$k_{et} = (\frac{1}{2RetCads})$

Table S3. The value of best fits to the original EIS data presented in (Figure 7c). The EIS data were interpreted by curve fitting the data to Randles circuit.

**SAM**	**R_{sol} (Ω)**	**C_{dl} (F)**	**R_{et} (Ω)**	**C_{ads} (F)**	**k_{et} (s^{-1})**
Cu (I) catalyst	6.52 ± 0.23	(9.99 ± 1.91)×10^{-6}	30.27 ± 0.57	(9.87 ± 0.21)×10^{-5}	167.36 ± 6.52
Ru (II) catalyst	4.13 ± 0.66	(1.01 ± 0.12)×10^{-6}	4000 ± 31.52	(2.51 ± 0.08)×10^{-6}	48.83 ± 1.94

Cartesian Coordinates of Optimized Structures

1,4 TS

B97D3/def2-SVPP

E_{DFT}: -2203.99733931

C	6.091936000	-0.217820000	-0.435306000
C	5.537555000	0.811849000	0.398932000
C	5.080229000	0.198907000	1.615597000
C	5.351935000	-1.209283000	1.531785000
C	5.978125000	-1.466000000	0.265307000
H	6.506424000	-0.078639000	-1.438828000
H	5.459713000	1.873722000	0.143986000
H	4.592762000	0.711231000	2.451333000
H	5.104504000	-1.958214000	2.290793000
H	6.289804000	-2.445407000	-0.111256000
Fe	4.124340000	-0.617024000	-0.003773000
C	2.324192000	0.264866000	-0.313755000
C	2.155043000	-0.957030000	0.430268000
C	2.678298000	-2.035975000	-0.356856000
C	3.157870000	-1.489531000	-1.596063000
C	2.930187000	-0.073789000	-1.574882000
H	1.724556000	-1.034310000	1.433860000
H	2.728351000	-3.087148000	-0.055614000
H	3.636994000	-2.052253000	-2.403494000
H	3.194820000	0.637379000	-2.363821000
C	1.882467000	1.631460000	0.122364000
H	1.935386000	1.709213000	1.229173000
H	2.560982000	2.399610000	-0.305057000
N	0.511156000	1.855320000	-0.349741000
C	-1.146833000	0.425734000	-0.463015000
C	-2.161212000	1.103723000	-0.197093000
H	-0.501429000	-0.393637000	-0.743094000
N	-0.171555000	2.819708000	0.063203000
N	-1.295265000	3.123070000	0.225829000
C	-3.585255000	1.417747000	0.020286000
H	-3.714542000	1.828121000	1.044008000
H	-3.884088000	2.243340000	-0.659569000
C	-4.515966000	0.206097000	-0.183207000
H	-4.374788000	-0.188927000	-1.210968000
H	-4.206111000	-0.607130000	0.506006000
C	-5.994094000	0.546732000	0.045126000
H	-6.294299000	1.366223000	-0.644143000
H	-6.124560000	0.950366000	1.073086000
C	-6.932841000	-0.650854000	-0.151811000
H	-6.639856000	-1.472150000	0.536148000
H	-6.806551000	-1.052926000	-1.180558000
C	-8.418163000	-0.307918000	0.082636000
H	-8.699724000	0.521378000	-0.604751000
H	-8.552382000	0.078369000	1.114738000
C	-9.334786000	-1.479421000	-0.148145000
H	-9.326889000	-1.897871000	-1.170931000

C	-10.118840000	-2.053136000	0.780148000
H	-10.155640000	-1.672988000	1.814827000
H	-10.755700000	-2.919537000	0.543300000

1,4 Model System & Gold Electrodes
86
symmetry c1

Au	0.000000000	0.000000000	0.000000000
Au	0.000000000	2.883753000	0.000000000
Au	2.883753000	0.000000000	0.000000000
Au	-2.883753000	0.000000000	0.000000000
Au	0.000000000	-2.883753000	0.000000000
Au	-1.442161000	1.442161000	2.039130000
Au	1.442161000	-1.442161000	2.039130000
Au	1.442161000	1.442161000	2.039130000
Au	-1.442161000	-1.442161000	2.039130000
Au	0.000000000	0.000000000	4.078260000
Au	0.000000000	2.883753000	4.078260000
Au	2.883753000	0.000000000	4.078260000
Au	-2.883753000	0.000000000	4.078260000
Au	0.000000000	-2.883753000	4.078260000
Au	-1.442161000	1.442161000	6.117390000
Au	1.442161000	-1.442161000	6.117390000
Au	1.442161000	1.442161000	6.117390000
Au	-1.442161000	-1.442161000	6.117390000
Au	0.000000000	0.000000000	8.156520000
Au	0.000000000	2.883753000	8.156520000
Au	2.883753000	0.000000000	8.156520000
Au	-2.883753000	0.000000000	8.156520000
Au	0.000000000	-2.883753000	8.156520000
Au	-1.442161000	1.442161000	10.195650000
Au	1.442161000	-1.442161000	10.195650000
Au	1.442161000	1.442161000	10.195650000
Au	-1.442161000	-1.442161000	10.195650000
Au	0.000000000	0.000000000	12.234780000
Au	0.000000000	2.883753000	12.234780000
Au	2.883753000	0.000000000	12.234780000
Au	-2.883753000	0.000000000	12.234780000
Au	0.000000000	-2.883753000	12.234780000
Au	-1.442161000	1.442161000	14.273910000
Au	1.442161000	-1.442161000	14.273910000
Au	1.442161000	1.442161000	14.273910000
Au	-1.442161000	-1.442161000	14.273910000
Au	0.000000000	0.000000000	16.313040000
C	0.008785000	0.142276000	23.835767000
C	0.076784000	0.977897000	24.921187000
N	0.020389000	0.963231000	22.750849000
H	-0.045214000	-0.926698000	23.748638000
N	0.097083000	2.288788000	23.173342000
N	0.127741000	2.279316000	24.444942000
C	-0.029404000	0.637340000	21.451223000
C	0.093418000	0.655227000	26.292552000
C	0.121885000	0.381377000	27.469637000
C	-0.090334000	0.378591000	20.274616000

S	0.000000000	0.000000000	29.102755000
S	0.000000000	0.000000000	18.643040000
Au	0.000000000	0.000000000	31.432760000
Au	-1.442161000	1.442161000	33.471890000
Au	1.442161000	-1.442161000	33.471890000
Au	1.442161000	1.442161000	33.471890000
Au	-1.442161000	-1.442161000	33.471890000
Au	0.000000000	0.000000000	35.511020000
Au	0.000000000	2.883753000	35.511020000
Au	2.883753000	0.000000000	35.511020000
Au	-2.883753000	0.000000000	35.511020000
Au	0.000000000	-2.883753000	35.511020000
Au	-1.442161000	1.442161000	37.550150000
Au	1.442161000	-1.442161000	37.550150000
Au	1.442161000	1.442161000	37.550150000
Au	-1.442161000	-1.442161000	37.550150000
Au	0.000000000	0.000000000	39.589280000
Au	0.000000000	2.883753000	39.589280000
Au	-2.883753000	0.000000000	39.589280000
Au	0.000000000	-2.883753000	39.589280000
Au	2.883753000	0.000000000	39.589280000
Au	-1.442161000	1.442161000	41.628410000
Au	1.442161000	-1.442161000	41.628410000
Au	1.442161000	1.442161000	41.628410000
Au	-1.442161000	-1.442161000	41.628410000
Au	0.000000000	0.000000000	43.667540000
Au	0.000000000	2.883753000	43.667540000
Au	2.883753000	0.000000000	43.667540000
Au	-2.883753000	0.000000000	43.667540000
Au	0.000000000	-2.883753000	43.667540000
Au	-1.442161000	1.442161000	45.706670000
Au	1.442161000	-1.442161000	45.706670000
Au	1.442161000	1.442161000	45.706670000
Au	-1.442161000	-1.442161000	45.706670000
Au	0.000000000	0.000000000	47.745800000
Au	0.000000000	2.883753000	47.745800000
Au	2.883753000	0.000000000	47.745800000
Au	-2.883753000	0.000000000	47.745800000
Au	0.000000000	-2.883753000	47.745800000

1,5 Model System & Gold Electrodes
86
symmetry c1

Au	0.000000000	0.000000000	0.000000000
Au	0.000000000	2.883753000	0.000000000
Au	2.883753000	0.000000000	0.000000000
Au	-2.883753000	0.000000000	0.000000000
Au	0.000000000	-2.883753000	0.000000000
Au	-1.442161000	1.442161000	2.039130000
Au	1.442161000	-1.442161000	2.039130000
Au	1.442161000	1.442161000	2.039130000
Au	-1.442161000	-1.442161000	2.039130000
Au	0.000000000	0.000000000	4.078260000
Au	0.000000000	2.883753000	4.078260000
Au	2.883753000	0.000000000	4.078260000
Au	-2.883753000	0.000000000	4.078260000
Au	0.000000000	-2.883753000	4.078260000
Au	-1.442161000	1.442161000	6.117390000
Au	1.442161000	-1.442161000	6.117390000
Au	1.442161000	1.442161000	6.117390000
Au	-1.442161000	-1.442161000	6.117390000
Au	0.000000000	0.000000000	8.156520000
Au	0.000000000	2.883753000	8.156520000
Au	2.883753000	0.000000000	8.156520000
Au	-2.883753000	0.000000000	8.156520000
Au	0.000000000	-2.883753000	8.156520000
Au	-1.442161000	1.442161000	10.195650000
Au	1.442161000	-1.442161000	10.195650000
Au	1.442161000	1.442161000	10.195650000
Au	-1.442161000	-1.442161000	10.195650000
Au	0.000000000	0.000000000	12.234780000
Au	0.000000000	2.883753000	12.234780000
Au	2.883753000	0.000000000	12.234780000
Au	-2.883753000	0.000000000	12.234780000
Au	0.000000000	-2.883753000	12.234780000
Au	-1.442161000	1.442161000	14.273910000
Au	1.442161000	-1.442161000	14.273910000
Au	1.442161000	1.442161000	14.273910000
Au	-1.442161000	-1.442161000	14.273910000
Au	0.000000000	0.000000000	16.313040000
C	3.276123000	1.030303000	21.208053000
C	4.562467000	1.433607000	20.938638000
N	3.244800000	0.959319000	22.591976000
H	5.022194000	1.614762000	19.983718000
N	4.471445000	1.312541000	23.103046000
N	5.237694000	1.587112000	22.108334000
C	2.221738000	0.614407000	23.385866000
C	2.186070000	0.729645000	20.381410000
C	1.243816000	0.485370000	19.663003000
C	1.311629000	0.287753000	24.106290000

S	0.000000000	0.000000000	18.643040000
S	0.000000000	0.000000000	25.111516000
Au	0.000000000	0.000000000	27.441520000
Au	-1.442161000	1.442161000	29.480650000
Au	1.442161000	-1.442161000	29.480650000
Au	1.442161000	1.442161000	29.480650000
Au	-1.442161000	-1.442161000	29.480650000
Au	0.000000000	0.000000000	31.519780000
Au	0.000000000	2.883753000	31.519780000
Au	2.883753000	0.000000000	31.519780000
Au	-2.883753000	0.000000000	31.519780000
Au	0.000000000	-2.883753000	31.519780000
Au	-1.442161000	1.442161000	33.558910000
Au	1.442161000	-1.442161000	33.558910000
Au	1.442161000	1.442161000	33.558910000
Au	-1.442161000	-1.442161000	33.558910000
Au	0.000000000	0.000000000	35.598040000
Au	0.000000000	2.883753000	35.598040000
Au	-2.883753000	0.000000000	35.598040000
Au	0.000000000	-2.883753000	35.598040000
Au	2.883753000	0.000000000	35.598040000
Au	-1.442161000	1.442161000	37.637170000
Au	1.442161000	-1.442161000	37.637170000
Au	1.442161000	1.442161000	37.637170000
Au	-1.442161000	-1.442161000	37.637170000
Au	0.000000000	0.000000000	39.676300000
Au	0.000000000	2.883753000	39.676300000
Au	2.883753000	0.000000000	39.676300000
Au	-2.883753000	0.000000000	39.676300000
Au	0.000000000	-2.883753000	39.676300000
Au	-1.442161000	1.442161000	41.715430000
Au	1.442161000	-1.442161000	41.715430000
Au	1.442161000	1.442161000	41.715430000
Au	-1.442161000	-1.442161000	41.715430000
Au	0.000000000	0.000000000	43.754560000
Au	0.000000000	2.883753000	43.754560000
Au	2.883753000	0.000000000	43.754560000
Au	-2.883753000	0.000000000	43.754560000
Au	0.000000000	-2.883753000	43.754560000

Nonadiyne Decorated Silicon

Si	4.22849589	4.19703233	-1.22340321
Si	2.29195015	3.08371150	1.98099781
Si	0.34889039	4.19636331	-1.16314714
Si	0.34529010	1.96570675	-1.96214893
H	0.34327471	1.99444032	-3.47494864
Si	2.28761878	0.83946220	-1.20732832
Si	2.28845079	0.85262839	1.15953521
Si	0.34464736	4.20029222	1.20937878
Si	2.27772571	5.31499770	-1.96940792
H	2.24238207	5.30555234	-3.48207818
Si	4.21323356	4.19670043	1.14144133
Si	4.23009311	1.95321085	-1.98363281
H	4.23102889	1.94421380	-3.49663921
Si	0.34451770	6.45631740	1.94073352
H	0.33773237	6.49093106	3.45461914
Si	4.22455480	6.43410037	1.92002915
H	4.23036873	6.42493595	3.43427608
Si	6.14938213	3.08121072	1.92420109
H	6.09895939	3.06570525	3.43850019
Si	6.18498190	5.31208650	-1.96304919
H	6.21922389	5.30173741	-3.47589102
Si	6.16848895	0.83912492	1.15951444
Si	6.17328518	0.83898716	-1.20745033
C	-1.61342700	-1.82190004	10.91987991
C	-0.95887965	-0.84313063	10.58630260
H	-2.19912346	-2.67616784	11.22884699
C	-0.18369255	0.32071244	10.16206317
H	-0.54322522	1.22073225	10.71080218
H	0.87576235	0.18637864	10.47733549
C	-0.23850035	0.58667859	8.64344538
H	0.10945644	-0.32343560	8.10705322
H	-1.29759846	0.73178490	8.33897905
C	0.60128117	1.79313400	8.21277608
H	1.65603565	1.63953532	8.53640093
H	0.25050121	2.70474707	8.74792844
C	0.56690450	2.03512016	6.69999538
H	-0.47708297	2.25061060	6.37969956
H	0.85582873	1.10201893	6.16726841
C	1.49457397	3.17313699	6.22727357
H	2.53892911	2.95172080	6.53725851
H	1.19882309	4.11588008	6.74276735
C	1.43429018	3.36813355	4.73707730
H	0.48852228	3.79456882	4.34974456
C	2.39957930	3.00156647	3.86122296
H	3.33224319	2.56668734	4.27486567
Tv	7.77102237	0.00000000	0.00000000
Tv	-0.00072861	6.71843568	0.00000000

1,4-Isomer tight-packed on Silicon

Si	3.64120494	3.81945346	0.83343441
Si	1.69378089	2.70273974	4.03639710
Si	-0.24162875	3.82056165	0.85000134
Si	-0.23582355	1.57682484	0.09626444
H	-0.21296564	1.55735515	-1.41654440
Si	1.69902753	0.46581024	0.89595650
Si	1.69952882	0.45930440	3.26953686
Si	-0.24215680	3.80753530	3.21662055
Si	1.69739843	4.95073882	0.09269677
H	1.68778004	4.98736139	-1.41995857
Si	3.62963559	3.80296304	3.19822133
Si	3.63432468	1.57274043	0.08998397
H	3.61239722	1.54151019	-1.42261792
Si	-0.24856819	6.04299724	3.99827149
H	-0.27782658	6.02831745	5.51274334
Si	3.65417071	6.03436792	3.98656261
H	3.69071055	5.99796567	5.50035168
Si	5.57424528	2.69877335	3.98475769
H	5.56917160	2.70592440	5.49819707
Si	5.58614299	4.93914436	0.07473697
H	5.59412635	4.94816161	-1.43833045
Si	5.58240106	0.45670798	3.22310605
Si	5.58249454	0.46034688	0.85437072
C	-6.71731660	0.61117556	18.76151512
C	-6.18081409	-0.70401719	18.52155508
C	-4.97864053	-0.83409734	19.30459270
C	-4.77171037	0.39681728	20.02155763
C	-5.84905466	1.29060984	19.68817703
H	-7.61366705	1.03586069	18.29167894
H	-6.60358504	-1.44884851	17.83302943
H	-4.32644660	-1.71628581	19.34945237
H	-3.93686517	0.61595704	20.69930419
H	-5.97810353	2.31529351	20.05996753
Fe	-4.83445886	0.74258308	18.00528339
C	-3.27209336	0.39796398	16.75174114
C	-3.08892503	1.63929084	17.47399289
C	-4.20349562	2.49600237	17.17061868
C	-5.07875443	1.79336765	16.27005957
C	-4.50671570	0.50159507	16.00607280
H	-2.24480265	1.87939883	18.13430495
H	-4.38017427	3.50076175	17.57350361
H	-6.02837194	2.17642193	15.87495244
H	-4.95711831	-0.28422531	15.38224705
C	-2.29822330	-0.73936725	16.69276491
H	-1.78573211	-0.87700287	17.66697901
H	-2.80945512	-1.69087399	16.44554767
N	-1.25658935	-0.52035346	15.67195654
C	-1.05320877	0.56150885	14.86725871
C	0.09220617	0.24449813	14.13881806

H	-1.69949524	1.44333160	14.88552327
N	-0.30534283	-1.45765937	15.46647165
N	0.50757845	-0.99984505	14.54143056
C	0.82462534	1.05480454	13.11165188
H	0.59470684	2.13031555	13.26964016
H	1.91426747	0.94276350	13.29805232
C	0.53910861	0.68172517	11.64467747
H	0.73758568	-0.40293006	11.49947002
H	-0.54155543	0.82984535	11.42579232
C	1.39175975	1.50972940	10.67827719
H	2.46772324	1.33396195	10.90797622
H	1.22273494	2.59178969	10.88300032
C	1.15087076	1.24279848	9.18892357
H	0.09250775	1.46555670	8.92872325
H	1.29725685	0.16087435	8.97008656
C	2.08641052	2.08008830	8.29865816
H	3.13817458	1.82098150	8.56796614
H	1.96864996	3.15952599	8.53901396
C	1.92250202	1.87589935	6.81864468
H	1.98403763	0.82650950	6.46925196
C	1.75052186	2.86731897	5.91280364
H	1.69544132	3.90603172	6.29819265
Tv	7.76644982	0.00000000	0.00000000
Tv	0.00330422	6.71534922	0.00000000

1,5-Isomer tightly-packed on Silicon

Si	5.10402056	3.15064385	-1.65539811
Si	3.16371846	2.03247370	1.49365317
Si	1.22502028	3.15029074	-1.70185591
Si	1.21802298	0.91277009	-2.47576029
H	1.21588828	0.91385723	-3.98869649
Si	3.15909677	-0.20248609	-1.70423746
Si	3.16009556	-0.19082339	0.66189770
Si	1.23875492	3.14749319	0.66467767
Si	3.17368090	4.26467382	-2.45929024
H	3.20091172	4.24851955	-3.97201871
Si	5.10667133	3.15432495	0.71670288
Si	5.10381978	0.92170857	-2.45590139
H	5.10803198	0.95156110	-3.96863711
Si	1.23682692	5.38751734	1.43137470
H	1.23528839	5.38634175	2.94617315
Si	5.11682762	5.41098972	1.44259595
H	5.12302929	5.45526486	2.95597211
Si	7.06762836	2.03736083	1.44252012
H	7.10740138	2.01798087	2.95651032
Si	7.04265312	4.26346656	-2.45035073
H	7.01585036	4.25074095	-3.96331555
Si	7.05279394	-0.19988116	0.66727874
Si	7.04624666	-0.20180417	-1.69948308
C	8.99624056	3.93093433	15.86842254
C	7.86551719	4.70663335	16.30623969
C	6.68860700	3.89256145	16.15788315
C	7.09054271	2.61494738	15.62964295
C	8.51949570	2.63824671	15.44969109
H	10.03982695	4.27003448	15.84444794
H	7.89538257	5.73917208	16.67759364
H	5.66130410	4.19211253	16.40389731
H	6.41621160	1.78287306	15.38761107
H	9.14015332	1.82897691	15.04230446
Fe	7.63459706	4.12006383	14.35972319
C	6.27928199	4.87485529	13.05174698
C	6.84788410	3.64562922	12.54180057
C	8.27380661	3.81753976	12.44570951
C	8.58662068	5.15607519	12.87804533
C	7.36265791	5.80976070	13.25065113
H	6.27747775	2.73603689	12.30947952
H	9.00312755	3.05522736	12.13644475
H	9.58469484	5.60534440	12.93577399
H	7.26744883	6.83934771	13.61951135
C	4.81894337	5.16149678	13.28052612
H	4.22349317	4.22978850	13.19380517
H	4.64398856	5.59563756	14.28344031
N	4.27549320	6.15930850	12.34995536
C	4.02641171	6.04231084	11.00863666
C	3.55744928	7.30947276	10.66398615

H	3.22847281	7.67864924	9.68687201
N	3.98895449	7.40957284	12.79815694
N	3.55163873	8.10335834	11.77464833
C	4.23610201	4.78779750	10.22167332
H	5.29729839	4.46995853	10.32595765
H	3.64945102	3.95947089	10.68130828
C	3.88719468	4.92484288	8.73426310
H	4.53529383	5.70438472	8.27477249
H	2.84294183	5.29226035	8.62532297
C	4.04645202	3.60761373	7.96813675
H	3.31105439	2.87026643	8.36153908
H	5.04793212	3.17290208	8.18762555
C	3.87747261	3.73084679	6.45057514
H	2.90159967	4.20797906	6.21059600
H	4.65488191	4.41752256	6.04606792
C	3.96696213	2.37345294	5.72702936
H	4.90555154	1.86752383	6.05542531
H	3.12987663	1.71579652	6.04766134
C	3.99162910	2.48180128	4.22854898
H	4.84001648	3.06209967	3.81574215
C	3.08943731	1.94185086	3.37568258
H	2.24486568	1.36846470	3.80838930
Tv	7.77026679	0.00000000	0.00000000
Tv	0.01313981	6.71044769	0.00000000

1,4-isomer sparsely-packed on Si

Si	5.22266375	3.13067140	-0.51412820
Si	3.28225803	2.01683508	2.68678868
Si	1.34225593	3.13105959	-0.51566297
Si	1.34848160	0.88271927	-1.26105883
H	1.37100343	0.85697589	-2.77377988
Si	3.28310232	-0.22710038	-0.45767692
Si	3.28360923	-0.22922428	1.91514959
Si	1.35213469	3.12396910	1.85086189
Si	3.28276478	4.25028533	-1.28770678
H	3.28346841	4.25064037	-2.80074459
Si	5.21339106	3.12395237	1.85296511
Si	5.21699763	0.88315123	-1.26193550
H	5.19319210	0.85966781	-2.77465463
Si	1.34121479	5.36720578	2.61753418
H	1.34220698	5.37375547	4.13155414
Si	5.22470296	5.36749215	2.61823177
H	5.22463254	5.37409119	4.13220262
Si	7.15840591	2.01072561	2.62890796
H	7.16095541	2.01463279	4.14197407
Si	7.16533165	4.24959975	-1.28292555
H	7.17022821	4.24797967	-2.79607325
Si	7.16619254	-0.23395534	1.86546544
Si	7.16474472	-0.23316837	-0.50290109
Si	12.98934630	3.13007133	-0.50751575
Si	11.04779475	2.00702842	2.62862379
Si	9.10571487	3.12979953	-0.50902681
Si	9.10534634	0.88736873	-1.27588086
H	9.10347150	0.88371470	-2.78898064
Si	11.04797902	-0.23286537	-0.50726640
Si	11.04845394	-0.23543372	1.86115853
Si	9.10405038	3.12527066	1.85926909
Si	11.04765610	4.24972577	-1.27820730
H	11.04838028	4.24753105	-2.79131452
Si	12.99172296	3.12585706	1.86086167
Si	12.99106728	0.88755507	-1.27406491
H	12.99467893	0.88376853	-2.78716169
Si	9.10610940	5.36821442	2.62449441
H	9.10373261	5.37271956	4.13796226
Si	12.98913593	5.36891115	2.62569140
H	12.99082064	5.37402474	4.13907789
Si	14.93764232	2.01150630	2.63041764
H	14.93751751	2.01774422	4.14373896
Si	14.92865903	4.25046835	-1.28282099
H	14.92208181	4.24988297	-2.79593933
Si	14.93073365	-0.23394970	1.86924090
Si	14.93103260	-0.23304117	-0.49928148
Si	5.22342418	9.85588933	-0.51375091
Si	3.28255669	8.72993943	2.62183077
Si	1.34074542	9.85607947	-0.51240282

Si	1.34129035	7.61504518	-1.28491004
H	1.33938200	7.61506338	-2.79801785
Si	3.28234726	6.49218235	-0.51760507
Si	3.28292424	6.48948838	1.85095070
Si	1.33984714	9.84998561	1.85473561
Si	3.28180209	10.99198824	-1.25882117
H	3.28127104	11.02647844	-2.77167030
Si	5.22525723	9.84888765	1.85330960
Si	5.22302902	7.61477167	-1.28580518
H	5.22442356	7.61437110	-2.79890658
Si	1.33302041	12.08407874	2.64261483
H	1.29951043	12.04918039	4.15539200
Si	5.23354597	12.08341410	2.63860454
H	5.26961415	12.04905996	4.15139622
Si	7.16574542	8.72910303	2.62406546
H	7.16371313	8.72817504	4.13760911
Si	7.16499881	10.97922914	-1.27785891
H	7.16648482	10.98337095	-2.79096068
Si	7.16598348	6.48893672	1.85247356
Si	7.16442429	6.49268177	-0.51594074
Si	12.98796271	9.85633545	-0.50886940
Si	11.04730838	8.73175132	2.62715222
Si	9.10582362	9.85609049	-0.51019115
Si	9.10649222	7.61465651	-1.28160306
H	9.10877913	7.61655238	-2.79471687
Si	11.04721622	6.49270023	-0.51157402
Si	11.04757286	6.49010380	1.85682527
Si	9.10626683	9.85375538	1.85829143
Si	11.04705788	10.97808588	-1.27800704
H	11.04733011	10.97854039	-2.79111576
Si	12.98860678	9.85400143	1.85962338
Si	12.98770939	7.61522024	-1.28096266
H	12.98559052	7.61739729	-2.79406274
Si	9.10693584	12.09520795	2.63002371
H	9.10921908	12.09246757	4.14349349
Si	12.98756560	12.09522799	2.63219764
H	12.98295437	12.09219977	4.14547997
Si	14.92924831	8.72980424	2.62629486
H	14.93114585	8.72896662	4.13956320
Si	14.92856450	10.97995387	-1.27596461
H	14.92660225	10.98563208	-2.78904526
Si	14.92906713	6.48984668	1.85368100
Si	14.92953644	6.49314579	-0.51474180
H	11.04647718	8.73140785	4.14052041
H	3.28345120	8.72921759	4.13543441
H	11.04722249	2.00786133	4.14206922
C	-7.84692200	-3.57471878	13.03117668
C	-6.74113162	-3.93866367	13.87772305
C	-6.53373874	-2.87033012	14.81984320
C	-7.51096287	-1.84736727	14.55560917

C	-8.32298168	-2.28327283	13.45123780
H	-8.25025634	-4.17206508	12.20375626
H	-6.15466687	-4.86379825	13.80971081
H	-5.76275936	-2.83982455	15.60075306
H	-7.61221377	-0.89700584	15.09500099
H	-9.15229607	-1.72400818	13.00000671
Fe	-6.35547489	-2.18633454	12.90212234
C	-4.41296767	-1.68145480	12.62677671
C	-5.25117628	-0.50443299	12.58677136
C	-6.21294512	-0.67687150	11.53228919
C	-5.96564536	-1.94982071	10.90770225
C	-4.85458035	-2.56841916	11.57602622
H	-5.17903161	0.35882147	13.26205693
H	-7.00547449	0.03146786	11.25948285
H	-6.53791262	-2.38114643	10.07659960
H	-4.41605391	-3.54736841	11.34316377
C	-3.25082473	-1.92530665	13.54850445
H	-3.34083255	-1.30235678	14.46182665
H	-3.20268115	-2.98656834	13.85884642
N	-1.95480121	-1.66355432	12.92142099
C	-1.41301251	-0.48062072	12.50926708
C	-0.18570240	-0.83715010	11.95570587
H	-1.92304274	0.47943780	12.63589284
N	-1.12592945	-2.69505927	12.63528360
N	-0.06360486	-2.20051219	12.05677193
C	0.87241302	0.00195651	11.30515192
H	0.71811714	1.06875292	11.57180374
H	1.85835090	-0.29128101	11.72686086
C	0.91761435	-0.15628534	9.77408125
H	1.00530916	-1.23858454	9.53615367
H	-0.05452498	0.17228877	9.34425598
C	2.06411233	0.61741365	9.11664987
H	3.03404236	0.25863619	9.53100174
H	1.99870892	1.69266318	9.40082352
C	2.09057858	0.50044305	7.58897969
H	1.13853447	0.89232660	7.16753908
H	2.12762547	-0.57387877	7.29958827
C	3.27271782	1.24488336	6.94002743
H	4.21993935	0.82541997	7.35331631
H	3.25289469	2.31782068	7.22991127
C	3.30398840	1.12188343	5.44303878
H	3.34676434	0.08482894	5.05565767
C	3.26766130	2.15203273	4.56485221
H	3.22165531	3.18040356	4.97699055
Tv	15.52937811	0.00000000	0.00000000
Tv	-0.00084207	13.45223899	0.00000000

1,5-isomer sparsely-packed on Si

Si	5.25043399	3.09483843	-1.29750112
Si	3.30807261	1.97147901	1.84848099
Si	1.37225062	3.09599296	-1.34865041
Si	1.37120132	0.85697585	-2.12389022
H	1.36928735	0.85905829	-3.63692344
Si	3.31110872	-0.26456279	-1.35400971
Si	3.31171731	-0.25337131	1.01288406
Si	1.38285030	3.09106138	1.01884840
Si	3.32093568	4.21462677	-2.09857599
H	3.35028629	4.20461995	-3.61137015
Si	5.25234824	3.09523316	1.07527831
Si	5.25476282	0.86479649	-2.10086831
H	5.26337327	0.89709427	-3.61361321
Si	1.37535624	5.33277739	1.79201340
H	1.37157171	5.33488558	3.30538438
Si	5.26276999	5.35361397	1.80265291
H	5.27117665	5.39962251	3.31580367
Si	7.21436133	1.97500171	1.79985663
H	7.26332818	1.96037817	3.31246246
Si	7.18457948	4.21264533	-2.09617990
H	7.15530758	4.19778188	-3.60905690
Si	7.19647666	-0.26511912	1.02643583
Si	7.19530380	-0.26377454	-1.34208736
C	6.70978089	3.60751871	17.44301242
C	5.41635094	4.22461218	17.30719392
C	4.54197169	3.28224203	16.65981248
C	5.29385955	2.08374798	16.39646511
C	6.63298855	2.28440821	16.88159319
H	7.60109020	4.06944826	17.88620699
H	5.14865894	5.23822872	17.63183852
H	3.48682811	3.44997538	16.40730339
H	4.91578671	1.18002514	15.90171265
H	7.45551911	1.56064467	16.81956365
Fe	6.15953881	3.69488544	15.47967696
C	5.48591322	4.73154549	13.87117065
C	5.95633168	3.42401561	13.47156476
C	7.33695791	3.30921644	13.85720369
C	7.72877487	4.54571573	14.48126343
C	6.59202193	5.42434367	14.48898185
H	5.35432777	2.64459129	12.98541565
H	7.97549425	2.42839753	13.71310944
H	8.71903886	4.77124655	14.89701688
H	6.55494179	6.44438803	14.89251389
C	4.11498333	5.31263758	13.64754312
H	3.38817715	4.51350586	13.39774645
H	3.75513593	5.83282621	14.55574577
N	4.08235283	6.33035056	12.59823819
C	4.10228749	6.18335194	11.23568340

C	4.11701358	7.50109089	10.77987951
H	4.12683218	7.87392823	9.74950469
N	4.09418079	7.64395776	12.95073455
N	4.11183750	8.34793550	11.85205889
C	4.09979219	4.86062850	10.53434426
H	4.99574121	4.27778466	10.84626021
H	3.22598579	4.25970181	10.87809459
C	4.06971201	4.96978981	9.00510517
H	4.94803912	5.56070032	8.66394530
H	3.17301543	5.54915011	8.69331099
C	4.06916869	3.60574952	8.30779655
H	3.18659761	3.01761153	8.64776739
H	4.95981237	3.02331668	8.63644171
C	4.06062483	3.69359411	6.77814429
H	3.17372380	4.27310856	6.43895182
H	4.94749126	4.27277893	6.43537836
C	4.05472162	2.31510267	6.09131594
H	4.93577814	1.73798877	6.45808605
H	3.15463459	1.73995170	6.39846310
C	4.12665404	2.39289962	4.59271062
H	5.01721868	2.91572906	4.19149735
C	3.21124084	1.89484399	3.72813377
H	2.32627139	1.37870065	4.15208331
Si	13.02157719	3.09871431	-1.35351215
Si	11.08532285	1.97883180	1.78606084
Si	9.13882086	3.09840784	-1.35139806
Si	9.13586542	0.85548135	-2.11629900
H	9.13268595	0.85296493	-3.62939852
Si	11.07892150	-0.26444352	-1.34983559
Si	11.08111753	-0.26347717	1.01878312
Si	9.14544802	3.10003373	1.01576102
Si	11.07947516	4.22037049	-2.12172572
H	11.08016092	4.22259083	-3.63481005
Si	13.02505107	3.09953501	1.01506478
Si	13.01990576	0.85630541	-2.12123967
H	13.01767413	0.85599294	-3.63435197
Si	9.14299128	5.34136105	1.78670050
H	9.14321174	5.33925904	3.30029165
Si	13.02574727	5.34013612	1.78674514
H	13.02304836	5.33739676	3.30011360
Si	14.96610161	1.97880997	1.78181388
H	14.95777391	1.97562898	3.29577977
Si	14.96497004	4.21930281	-2.11931352
H	14.96573319	4.22279318	-3.63246363
Si	14.96369507	-0.26365445	1.01458765
Si	14.96242129	-0.26441182	-1.35404239
Si	5.25625016	9.82352960	-1.34622521
Si	3.31649632	8.70520324	1.79055331
Si	1.37464057	9.82220672	-1.35136757
Si	1.37515688	7.57881164	-2.11580362

H	1.37915837	7.57450875	-3.62890375
Si	3.31549010	6.45951285	-1.34041858
Si	3.31542406	6.46139018	1.02812009
Si	1.37548749	9.82375190	1.01717352
Si	3.31629715	10.94328540	-2.12046500
H	3.31868466	10.93718856	-3.63360587
Si	5.25778809	9.82801285	1.02228589
Si	5.25501363	7.58124634	-2.11345580
H	5.25209024	7.57978824	-3.62655644
Si	1.37785734	12.06621963	1.78048473
H	1.37342065	12.06470504	3.29443830
Si	5.25169950	12.07039107	1.79031068
H	5.24885513	12.06758838	3.30322693
Si	7.20065754	8.70689878	1.78956242
H	7.20215826	8.70576882	3.30297032
Si	7.19701325	10.94581609	-2.11503699
H	7.19650599	10.94888016	-3.62816285
Si	7.20227360	6.46581549	1.01971775
Si	7.19763557	6.46083132	-1.34762740
Si	13.02352346	9.82318282	-1.34990370
Si	11.08429674	8.70329060	1.78766376
Si	9.13928898	9.82350580	-1.34922116
Si	9.13890818	7.58134649	-2.11862612
H	9.13911975	7.58193903	-3.63172563
Si	11.08136370	6.46125563	-1.35032578
Si	11.08454194	6.46193731	1.01836354
Si	9.14296783	9.82487429	1.01944614
Si	11.08135526	10.94382119	-2.11843697
H	11.08142328	10.94398141	-3.63154946
Si	13.02595402	9.82361625	1.01864967
Si	13.02404801	7.58127221	-2.11794063
H	13.02566525	7.58076518	-3.63104746
Si	9.14406286	12.06549092	1.79015267
H	9.14803261	12.06307935	3.30348998
Si	13.02680778	12.06586630	1.78591888
H	13.02819659	12.06712842	3.29927737
Si	14.96750254	8.70223807	1.78758944
H	14.96751987	8.70497672	3.30092937
Si	14.96509283	10.94311935	-2.12051916
H	14.96429203	10.94131594	-3.63363420
Si	14.96944464	6.45932619	1.02129087
Si	14.96499173	6.45978799	-1.34733296
H	11.08412691	8.70274457	3.30094490
H	3.31317151	8.70979490	3.30399260
H	11.08514617	1.98030180	3.29945689
Tv	15.53246541	0.00000000	0.00000000
Tv	0.00354301	13.45027297	0.00000000
